# Effect of exogenous treatment with zaxinone and its mimics on rice root microbiota across different growth stages

**DOI:** 10.1038/s41598-024-82833-6

**Published:** 2024-12-28

**Authors:** Teresa Mazzarella, Matteo Chialva, Leonardo Perez de Souza, Jian You Wang, Cristina Votta, Rhowell Tiozon, Patrizia Vaccino, Alessandra Salvioli di Fossalunga, Nese Sreenivasulu, Tadao Asami, Alisdair R. Fernie, Salim Al-Babili, Luisa Lanfranco, Valentina Fiorilli

**Affiliations:** 1 Department of Life Sciences and Systems Biology, University of Turin, Viale Mattioli 25, Turin, 10125 Turin Italy; 2 Max-Planck-Institute of Molecular Plant Physiology, Am Mühlenberg 1, 14476 Potsdam-Golm, Germany; 3 The BioActives Lab, Center for Desert Agriculture (CDA), Biological and Environment Science and Engineering (BESE), King Abdullah University of Science and Technology, Thuwal, 23955-6900 Saudi Arabia; 4 Consumer-driven Grain Quality and Nutrition, Rice Breeding Innovation Department, International Rice Research Institute, Los Baños, Philippines; 5 Council for Agricultural Research and Economics CREA-CI,—Research Centre for Cereal and Industrial Crops, s.s. 11 to Torino, km 2.5, Vercelli, 13100 VC Italy; 6 Graduate School of Agricultural and Life Sciences, The University of Tokyo, 1-1-1 Yayoi, Bunkyo-ku, Tokyo, 113-8657 Japan; 7 Division of Biological and Environmental Sciences and Engineering, King Abdullah University of Science and Technology (KAUST), Thuwal, 4700, 23955-6900 Kingdom of Saudi Arabia; 8 Centre of Excellence for Sustainable Food Security, King Abdullah University of Science and Technology, Thuwal, Kingdom of Saudi Arabia

**Keywords:** *Oryza sativa*, Zaxinone, MiZax, Microbial communities, Rhizosphere, Shoot metabolism, Ecology, Microbiology, Plant sciences

## Abstract

Enhancing crops productivity to ensure food security is one of the major challenges encountering agriculture today. A promising solution is the use of biostimulants, which encompass molecules that enhance plant fitness, growth, and productivity. The regulatory metabolite zaxinone and its mimics (MiZax3 and MiZax5) showed promising results in improving the growth and yield of several crops. Here, the impact of their exogenous application on soil and rice root microbiota was investigated. Plants grown in native paddy soil were treated with zaxinone, MiZax3, and MiZax5 and the composition of bacterial and fungal communities in soil, rhizosphere, and endosphere at the tillering and the milky stage was assessed. Furthermore, shoot metabolome profile and nutrient content of the seeds were evaluated. Results show that treatment with zaxinone and its mimics predominantly influenced the root endosphere prokaryotic community, causing a partial depletion of plant-beneficial microbes at the tillering stage, followed by a recovery of the prokaryotic community structure during the milky stage. Our study provides new insights into the role of zaxinone and MiZax in the interplay between rice and its root-associated microbiota and paves the way for their practical application in the field as ecologically friendly biostimulants to enhance crop productivity.

**Supplementary Information:**

The online version contains supplementary material available at 10.1038/s41598-024-82833-6.

## Introduction

Ensuring food security for the world population is challenged by a variety of factors, including climate change, environmental pollution, and, in particular, the rapidly escalating demand to address the expanding human population^1^. According to the United Nations Food and Agriculture Organization (FAO) estimates, agriculture will need to increase food production by nearly 70% by 2050. There is a projected need for a 112% increase in food production to meet anticipated caloric requirements in specific regions such as South Asia and sub-Saharan Africa^1^.

Rice (*Oryza sativa* L.), a member of the *Poaceae *family, is a global major food crop, supplying staple sustenance to nearly half of the world’s population^2^. Approximately 60% of the world’s rice is cultivated in Southeast Asia, and its production and consumption are on the rise in Africa as well^3^. Currently, the productivity of rice is threatened by pests, soil degradation^4,5^, diminishing water^6^, and environmental pollution^7^. Weeds (37.02%), insects (27.9%), and fungal pests (15.6%) are recognized as primary contributors to yield losses^8^. In sub-Saharan Africa, parasitic weeds of the genus *Striga *cause significant yield reductions^9^, a situation expected to worsen with the influence of climate change^10^. Given that modeling projections for rice production indicate an emerging constraint on yields^11^, global adaptation and mitigation strategies are imperative.

A promising solution is the use of growth-promoting biostimulants that include molecules and/or microorganisms enhancing plant fitness in terms of plant growth, productivity, and nutrient utilization efficiency. Additionally, biostimulants may have the capacity to bolster tolerance against a broad spectrum of abiotic and biotic stresses^12–14^.

The employment of plant-associated microorganisms (plant microbiota) represents a particularly promising, long-term solution to the challenges of attaining food security and preserving the environment^15^. The plant microbiota is shaped by eco-evolutionary processes^16^driven by the metabolic affinity between partners and chemical signals released by plant roots into the rhizosphere to screen the microbial community^17,18^.

Besides beneficial microbes, the application of phytohormones or hormone-like compounds, such as auxins and cytokinins or sterols and polyamines, respectively, showed positive effects on plants, including the promotion of plant growth and productivity^19^.

The plant pigments carotenoids are a source of a series of regulatory metabolites. Oxidative cleavage of these carotenoids leads to a class of compounds called apocarotenoids that encompass precursors for the phytohormones abscisic acid (ABA) and strigolactones (SLs), as well as bioactive metabolites and growth regulators, such as β-cyclocitral, anchorene, and zaxinone^20^. Indeed, apocarotenoids play a role in nearly all aspects of plant physiology and development, contribute to the plant response to both abiotic and biotic stresses, and mediate plant-plant and plant-microbe interactions^20^.

The carotenoid-derived hormone SLs regulates various aspects of plant development, including shoot branching, root architecture, and leaf senescence, and modulates plant responses to both abiotic and biotic stress^21–24^. Additionally, SLs play a pivotal role in rhizospheric communications, manifesting both negative and positive effects^25,26^. On one hand, they induce the germination of seeds of root parasitic plants, which is followed by infestation that causes substantial yield losses in numerous crops^27,28^. On the other hand, they act as chemical signals attracting arbuscular mycorrhizal (AM) fungi and facilitating the establishment of beneficial AM symbiosis^29^. Recent studies have indicated that SLs also influence the composition of the rhizosphere microbial community^26,30^and regulate plant-pathogen interactions^23,31^.

Zaxinone is emerging as a crucial regulator of rice growth, metabolism, hormone homeostasis, and AM symbiosis^32,33^. Its plant growth-promoting effect is mediated by an enhancement of root sugar uptake and metabolism, and a modulation of SL and cytokinin content^34,35^. The limited availability of zaxinone, due to a complex labor-intensive organic synthesis, has been overcome by the development of easily synthesizable and highly efficient zaxinone mimics (MiZax)^36^. MiZax3 and MiZax5 exhibit zaxinone-like activities, such as rescuing root growth in zaxinone-deficient rice mutants, promoting overall growth, and reducing SL content in wild-type plants. Exogenous applications of zaxinone, MiZax3, and MiZax5 demonstrated their utility and growth-promoting effects on rice and various horticultural crops under both normal and desert conditions^37,38^. Additionally, these compounds have the ability to alleviate the infestation by the root parasitic plant Striga by decreasing SL biosynthesis^33,37,38^. Interestingly, MiZax compounds were at least as efficient as zaxinone in reducing Striga infestation and had no negative impact on mycorrhization^38^. These data highlight that zaxinone, and in particular, the highly efficient MiZax are excellent biostimulants and helpful tools for establishing sustainable agriculture and alleviating the infestation by parasitic plants. However, their impact on soil microbial community composition is still unknown. With the aim to promote the use of these novel growth-promoting compounds as biostimulants, we investigated whether the exogenous application of zaxinone or MiZax(s) on the soil could influence soil microbiota communities, the recruitment of rice root-associated microbes, and shoot and grain metabolism.

Our results show that treatment with zaxinone and MiZax mostly impacted the prokaryotic component of the root endosphere. However, network analysis highlighted a partial perturbation of taxa-taxa interactions at the vegetative stage (tillering), followed by a full recovery of a complex network, structured by relevant beneficial microbial hubs, during the fruit set (milky stage). Using microbial ecology tools, we provide here new insights into the role of zaxinone and MiZax in the interplay between plants and rice root-associated microbiota.

## Materials and methods

### Plant growth conditions, hormonal treatments, and sampling

The impact of zaxinone, MiZax3, and MiZax5^36^ on native paddy soil and rice rhizomicrobiota was studied in a greenhouse mesocosm experiment. Four soil treatments were considered, namely zaxinone, MiZax3, MiZax5, and the solvent acetone as control (ACE), for both rice plants and unplanted soil. Plants were sampled at three phenological stages, the tillering stage (60 days after transplanting, T1) the milky-stage maturation (120 days after transplanting, T2), and the over-ripe stage (180 days after transplanting, T3). For each treatment a total of 33 plants were grown: 15 for phenotyping sampled at T3 and 18 for microbiota profiling (sampled at T1 and T2, 9 biological replicates each). For the unplanted soil treatment, 18 replicates were collected (9 for each sampling point).

Mesocosm systems were set up using native paddy soil harvested from the experimental fields of the CREA-CI research center (Vercelli, Italy) which was used in previous studies^39,40^. Soil physico-chemical parameters measured on a representative batch used for this study are reported in Table S1. Rice seeds (*Oryza sativa* cv. ‘Nipponbare’) were sown in alveolar trays filled with soil. After 1 month of growth under controlled conditions, plants were transferred to the final plastic pots (10 × 9 × 17 cm) filled with the same soil. For unplanted soil treatment, one alveolar tray was left unsown and the resulting soil cores were transferred into pots with fresh soils (as for rice seedlings) for the unplanted soil conditions. Plants and unplanted soils were grown in the greenhouse at the Department of Life Sciences and Systems Biology of the University of Torino from June 2021 to October 2021 (rice growing season) without monitoring light, temperature and humidity. Plants were watered once a week with tap water and once with distilled water containing zaxinone, MiZax3, and MiZax5 molecules dissolved individually to reach the final concentration of 5µM (10^−6^), which has already been shown to promote growth activity in rice plant and to alleviate infestation by the root parasitic plant Striga^35,36^. Fifty mL of the solution was poured into the soil of each pot once every two weeks for about 5 months (10 treatments) to cover the vegetative and rice reproductive growth stages.

### Compartment isolation and microbiota profiling

Plants were sampled for microbiota profiling at the tillering stage (T1) and at the milky-stage maturation (T2). At sampling, plants were removed from pots, vigorously shaken, and 10–15 g of roots collected within 3–4 cm from the base of the stem into a 50-mL Falcon tube. Unplanted soil samples were collected using a sterile spoon from the middle core of the pot, discarding edges that were in contact with plastic or any other portions where plant roots were visible. Samples were stored at + 4 °C and processed within 24 h to separate plant root compartments under sterile conditions. Samples were then processed to isolate the rhizosphere from the root endosphere according to the protocol by Bulgarelli et al.^41^ with minor modifications. Roots fragments were first washed into 10 mM sterile phosphate-buffer saline with 0.02% Tween-80 added (PBS-T), under continuous stirring on a horizontal shaker (15 min, 70 rpm). To obtain the rhizosphere soil slurry, roots were removed and tubes were centrifuged (4000 g, 10 min). The rhizosphere was then resuspended in 2 mL PBS-T and snap-freezed in liquid nitrogen. Roots were enriched in the endospheric compartment by two washes of 10 sonication cycles (30 s pulses, 30 s rest each) in PBS-T, discarding and replacing the buffer after each wash. Samples were finally rinsed in 50 mL of sterile dH_2_0, blotted on sterile filter paper, and stored at −80 °C until DNA isolation. For each time point, at least two aliquots of the same PBS-T buffer used to wash samples were collected and further processed along samples as blanks.

DNA was extracted under sterile conditions from 0.5 g of unplanted soil or 500 µL of rhizospheric soil slurry with the NucleoSpin Soil kit (Macherey-Nagel, Düren, Germany) and from 20 mg freeze-dried root material using the NucleoSpin Plant II Mini kit (Macherey-Nagel) following manufacturer’s recommendations. DNA quantity and purity were assessed using a Nanodrop-1000 instrument (Thermo Scientific, Wilmington, Germany). DNA materials were sent for gene marker amplification and sequencing to IGA Technology Services (Udine, Italy; http://igatechnology.com/). For Prokaryotic communities profiling the V4 16S region was selected using primers pairs 515F (5’-GTGYCAGCMGCCGCGGTAA-3’) and 806R (5’-GACTACNVGGGTWTCTAAT-3’)^42,43 ^linked with the Illumina adapters overhang. Amplification on organellar rDNA was prevented by peptide nucleic acids (PNAs) clamping using universal pPNA and mPNA clamps for plastidial and mitochondrial sequences following the manufacturer’s protocol (PNA Bio Inc, Newbury Park, CA). For fungi, the ITS2 region of the rRNA gene was adopted as marker using primers pair fTIS7 (5’-GTGARTCATCGAATCTTTG-3’) and ITS4 (5’-TCCTCCGCTTATTGATATGC-3’)^44^. Libraries from both target regions (16 S and ITS2) were then constructed and sequenced on an Illumina NovaSeq6000 platform (Illumina, San Diego, CA, USA) with a 2 × 250 bp sequencing layout.

### Bioinformatics

Amplicon libraries were inspected for quality using FastQC v0.11.9^45 ^and multiQC v1.11 software^46 ^and raw reads imported into QIIME 2 (Quantitative Insights Into Microbial Ecology) v2022.02^47^ for denoising, Amplicon Sequence Variants (ASVs) detection and taxonomy mapping. First, primers were fully removed from reads using the cutadapt ‘trim-paired’ plugin^48 ^discarding untrimmed sequences. For ITS2 libraries the full-length ITS2 region was selected using ITSxpress plugin with the built-in fungal database to increase taxonomic resolution^49^. Clean reads were then denoised and merged into ASVs using DADA2 plugin^50 ^in ‘pooled’ chimera method detection and applying a reads truncation of 180 and 160 bp based on quality profiles for R1 and R2 sequences, respectively. No reads truncation was applied for ITS2 libraries (--p-trunc-len 0). Variants were then taxonomically annotated using a Naïve-Bayes classifier via the ‘feature-classifier classify-sklearn’ plugin^51^. The SILVA v138 database (99% clustering) pre-formatted for QIIME^52,53 ^and the UNITE + INSDC v8.3 database in developer and dynamic mode^54 ^were used as reference databases for 16S and ITS2 libraries, respectively. Tables were further taxonomy-filtered to obtain the final feature table analyzed. For the 16S dataset, ASVs matching organellar (mitochondria and chloroplast) rDNA, or without any match (unassigned at the domain level) were removed while for ITS2 libraries non-fungal sequences were discarded. The obtained feature tables was imported into R v4.2.1 environment (R Core Team, 2023) and contaminants were removed using the extraction-blank samples with the microDecon package^55^. Alpha-and beta-diversity analyses were performed using ‘phyloseq’ v1.40.0^56^, ‘vegan’ v2.6-2^57^, and ‘QsRutils’ v0.1.5^58^. The ASVs count table was first filtered by removing low-abundance ASVs using ‘HTSFilter’ v1.36.0^59 ^and then normalized with a rarefaction-free approach using DEseq2 v1.36.0^56,60^.

Analyses of *β-*diversity were performed on the resulting normalized table. PERMANOVA and pairwise PERMANOVA analyses were performed using the *adonis *function of the R package ‘vegan’ and the package ‘pairwiseAdonis’ v0.4.1^61^, respectively. Principal coordinate analysis (PCoA) was performed by multidimensional scaling (MDS) of Bray–Curtis distance matrices using *cmdscale* R function. Constrained ordination (cPCoA) were computed using the ‘vegan’ *capscale *function (which implements CAP (Canonical analysis of principal coordinates)^62^ by constraining the factor of interest. Significance of constraints was assessed using the ANOVA-like permutation test implemented in the *anova.cca* function from the vegan package (999 permutations, P < 0.05).

Compartment enrichment and differential abundance analyses were performined using DESeq2 package applying a zero-tolerant geometric mean formula, as detailed in phyloseq package vignettes, and adopting an FDR threshold of 0.05 to define enriched/depleted taxa. Phylogenetic heatmaps were obtained using the ggtreeExtra R package^63 ^keeping only highly-abundant taxa (relative abundance > 5%) annotated at least at family level. Briefly, ASV sequences of differentially abundant taxa were aligned using MUSCLE (default parameters), and approximately-ML phylogenetic trees were obtained using FastTree v2.1.11^64^ using the GTR model and enabling ‘-no2nd’ option and setting SPRs number as 4.

Graphical elaborations were performed using ‘ggtern’ v3.3.5^65 ^and ggplot2 v3.3.6^66^ packages.

### Network analysis

Co-abundance networks of the root endosphere prokaryotic community for each treatment and time points were inferred using network analysis. The most abundant taxa (occurring in > 50% of the samples with at least 350 reads across all the considered samples) were selected for each of the subsampled tables and co-abundance networks inferred using the SPIEC-EASI (Sparse Inverse Covariance Estimation for Ecological Association and Statistical Inference) algorithm in SpiecEasi R package v1.1.2^67 ^using the Meinshausen and Bühlmann neighborhood selection model, a lambda path of 100 and other parameters at default values. The final model was selected by random subsampling and interaction re-estimation using stability Approach to Regularization Selection (StARS) and pulsar packages using 100 random subsamples at 0.05% variability threshold. The obtained adjacency matrices were imported into igraph objects and networks plotted using ggraph R package v2.1.0^68^. Network statistics and node’s topological parameters were calculated using igraph R package v1.5.1^69^ and differences across conditions were assessed using the Student’s *t*-test (*P* < 0.05).

For each of the obtained networks, 1000 re-wired random networks (same number of nodes and edges as the real ones) were obtained using the Erdős–Rényi model^70^ with the ‘sample_gnm’ function in igraph and metrics calculated as detailed above. Differences of the topological metrics between random and real networks were assessed using the Z-test (*p*< 0.05) within the BSDA v1.2.2 R package^71^.

Keystone species (hubs-taxa) were identified as the top 5% ASVs showing maximum closeness centrality and betweenness centrality metrics according to a log-normal distribution^72,73^.

### Gas chromatography-mass spectrometry (GC-MS) analysis

Powdered shoot and root material (50 mg ± 10%) was extracted in 700 µL of methanol by adding 10 µg/mL of methyl α-D-glucopyranoside as internal standard. The samples were homogenized by vortexing and by using a Ball Mill (Retsch, MM 300) with 5 mm zirconia balls (3 min, 20 Hz) and then centrifuged (10 min, 21000 g) recovering 600 µL of the resulting supernatant. The sample was mixed by vortexing with 300 µL of chloroform and 750 µL of water and centrifuged (10 min, 21000 *g*). Aliquots of 100 µL and 300 µL from the polar phase were dried in a SpeedVac™ concentrator for GC-MS analysis, respectively. Samples for GC-MS were derivatized according to Lisec et al.^74^. The samples were re-suspended in 40 µL of methoxyaminhydrochloride (20 mg/mL in pyridine) and shaked for 2 h (37 C°, 900 rpm). After that, 70 µL MSTFA were added, and the samples were mixed for additional 30 min (37 C°) and transferred to a glass vial for GC-MS analysis.

GC-MS analysis was performed on an Agilent 7890A GC system coupled to a Pegasus HT high throughput TOF/MS (LECO). 1 µL of the sample was injected at 230 °C in splitless mode with He as a carrier gas (2 mL/min). The flow rate was kept constant with electronic pressure control enabled. Chromatography was performed in a 30 m MDN-35 capillary column, with the following temperature program: isothermal for 2 min at 80 °C, followed by a 15 °C per min ramp to 330 °C, and isothermal for 6 min at 330 °C. Transfer line and ion source temperatures were set to 250 °C. The recorded mass range was set from m/z 70 to m/z 600 at 20 scans per second. The remaining monitored chromatography time was preceded by a 170 s solvent delay with filaments turned off. The manual mass defect was set to filament bias current to − 70 V, and detector voltage to ~ 1700–1850 V.

The obtained chromatograms were converted to .raw file format and analyzed using the Xcalibur 2.2 software (Thermo Fisher). GC-MS peaks were annotated by comparing retention indexes relative to a mixture of fatty acid methyl esters (FAMES) and spectra similarity against metabolites from the Golm metabolome database (GMD)^75^. Statistical analysis was conducted using MetaboAnalyst software. Five different biological replicates were analyzed for each condition.

### Biochemical analyses

*Sample extraction*

The Pereira-Caro et al.^76^ method was adapted to simultaneously extract the target lipophilic components from rice seeds, with some modifications. Dehulled rice seeds collected from panicles were grounded using a TissueLyser (Retsch) machine (25 Hz, 2 min) to obtain 2 g for each genotype of rice flour from whole grain (brown rice) and extracted for 1 h in an ultrasonicator with 10 mL of ethanol/hexane (4:3, v/v) mixture containing 0.1% ascorbic acid (w/v). After homogenization, samples were centrifuged for 15 min (9000 rpm at 20 °C). The obtained pellets were re-extracted twice using 5 mL hexane, mixed by vortexing, sonicated for 1 h and centrifuged as described above. The resuspended pellet was pooled and washed first with 10 mL distilled water and 5 mL of 10% NaCl solution. The organic phase was retained and reduced to almost dryness using a rotary vacuum evaporator at 35 °C. A small amount of 90% ethanol was added to remove adhering residues on the wall. The concentrated extract was frozen at −20 °C, freeze-dried for 24 h and stored in the dark at 4 °C for further analyses. Three different biological replicates were analyzed for each condition.

*Antioxidant analysis*

The antioxidant assays such as DPPH (2,2-Diphenyl-1-picrylhydrazyl), FRAP (Ferric Reducing Antioxidant Power), and ABTS (2,2’-Azino-bis(3-ethylbenzothiazoline-6-sulfonic acid)) were performed using the previous method^77^. Briefly, Trolox in ethanol (serial dilutions) was used as a positive control, and a blank control was prepared. DPPH, FRAP, and ABTS are measured in 515 nm, 620 nm, and 734 nm, respectively. All antioxidant values were expressed as Trolox equivalents/100 g rice (µmol TE/100 g). Three different biological replicates were analyzed for each condition.

*Total starch quantification*

The rice seeds were dehulled and milled as described before and total starch spectrophotometrically-quantified using the Total Starch (AA/AMG) Assay Kit (Megazyme Ltd., Ireland), following the manufacturer’s instructions. Three different biological replicates were analyzed for each condition.

*Mineral determination*

The minerals were quantified in the rice seed samples using the Inductively Coupled Plasma Optical Emission spectroscopy (ICP-OES) following a previously published method^77^. From the digested samples of rice, twelve minerals such as Al, Ca, Cu, Fe, K, Mg, Mn, Mo, Na, P, S, and Zn were quantified.

### Statistical analysis

All the statistical analyses were performed in the R statistical environment (R Core Team, 2023). Data normality and homoscedasticity were tested using Shapiro–Wilk^78 ^and Levene’s test^79^ using the ‘stats’ and ‘car’ v3.1–2packages^80^, respectively (P < 0.05). According to data distributions, ANOVA for normal homoscedastic data or Kruskal–Wallis test for non-normal homoscedastic data^81 ^were applied (P < 0.05) using custom base R function or the ‘agricolae’ package ^82^. Multiple comparisons between treatments were performed using Tukey’s HSD or Dunn’s post-hoc tests after ANOVA or Kruskal-Wallis respectively (p < 0.05), using the package ‘agricolae’ v1.3^82 ^or ‘rstatix’ 0.7.2^83^. When comparing two experimental groups the Welch t-test was applied as implemented in package ‘ggpubr’ v0.6.0^83^. All data visualizations were performed in R using ‘ggplot2’ v3.3.6^66^.

## Results

### Zaxinone and its mimics shape soil and root prokaryotic communities but not mycobiota assembly

To investigate the impact of exogenous treatment with zaxinone and its mimic molecules (MiZax3 and MiZax5) on soil and plant-associated microbial communities, deep 16S and ITS2 rRNA gene amplicon sequencing on unplanted soil and rice rhizosphere and endosphere collected at two timepoints (tillering and milky stage) were performed. A total of ~ 40 and ~ 50 M high-quality reads for the *16S* and *ITS2* markers, were obtained respectively. After primers removal, sequence denoising, ASV calling, and removal of non-target sequences (plant organellar DNA and non-fungal sequences), a total of 20,875,118 and 20,600,037 fragments were retained and used for subsequent analyses for 16S and ITS2 markers, respectively (Dataset S1). A total of 31,797 16S ASVs (bASVs) and 2712 ITS2 ASVs (fASVs) were obtained, optimally covering diversity for both markers (Figure S1-S2).

**Fig. 1:**
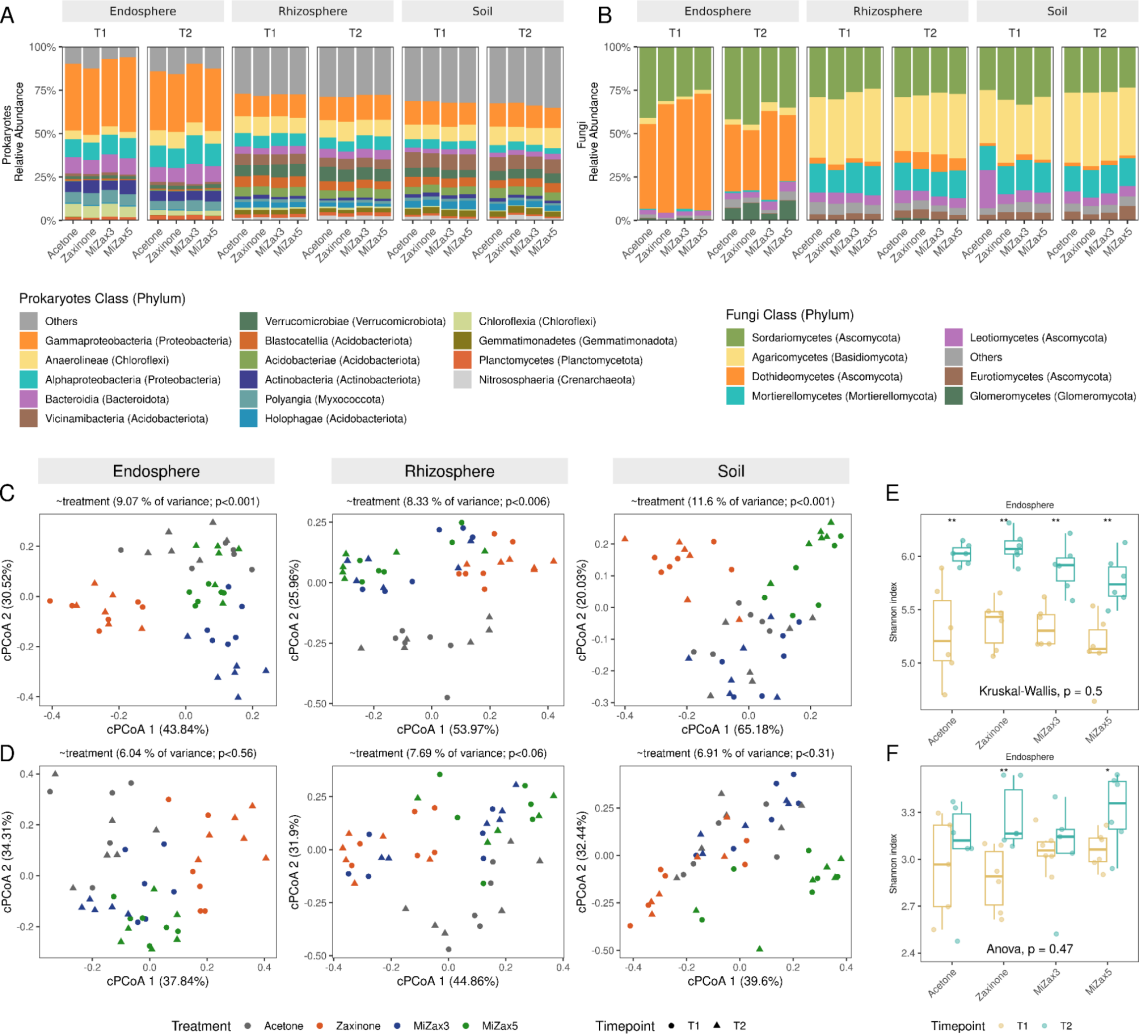
Assembly and diversity of rice (*Oryza sativa* ) root-associated microbiota under zaxinone and MiZax treatments at tillering (30 days after transplanting, T1) and milky-stage (90 days after transplanting, T2). (**A**-**B**) Average relative abundances of prokaryotes (A) and Fungi (B) at the Class level; the Phylum is indicated in brackets. (**C**-**D**) Constrained Principal Coordinate Analysis (cPCoA) plot of Bray–Curtis distances between samples by treatment of prokaryotic (C) and fungal communities (D) across the different compartments. Points represent single samples and are coloured by treatment. The fraction of the total variance explained by the projection is indicated in brackets along each axis. (**E**-**F**) *α*-diversity analysis (within sample diversity) based on Shannon Index of Prokaryotic and Fungal communities. Asterisks indicate significant differences between timepoints (T1 and T2) according to the Student’s *t*-test (* *P* < 0.05, ** *P* < 0.01, *** *P* < 0.001). No significant effect of the treatment was detected for both groups (Fungi and Prokaryotes) and compartments (unplanted soil and rhizosphere); the treatment p-value from Kruskal-Wallis/ANOVA analysis is indicated in the figure.

The root endospheric prokaryotic community was dominated by Proteobacteria, Myxococcota, Chloroflexi, Actinobacteriota, and Bacteroidota, while in rhizosphere and soil, a higher abundance of Acidobacteria and Planctomycetes was detected (Fig. 1A); this trend is in line with literature data describing the rice microbiota^84,85^. The root endophytic fungal community was dominated by Sordariomycetes and Dothideomycetes class (Ascomycota), while in rhizosphere and soil, there was a prevalence of Agaricomycetes (Basidiomycota) and Mortierellomycetes (Mortierellomycotina, Fig. 1B).

Principal Coordinate Analysis (PCoA) on both datasets showed that the influence of zaxinone and MiZax treatments was less marked compared to the effect of compartment factors, validating the robustness of the protocol used to collect rhizocompartments (Figure S3, Table S2). However, at least for prokaryotic communities, PERMANOVA analysis indicated a significant effect of treatments (*P* < 0.05, 9999 permutations; Table S2). Indeed, constraining PCoA ordinations by treatment factor using canonical analysis of principal coordinates^62^ in all compartments and timepoints, a clear separation of bacterial communities across treatments emerged (*P* < 0.05; Fig. 1C) with a closer clustering of Prokaryotic communities in plants/soils treated with MiZax3 and MiZax5 and a neat separation of zaxinone-treated samples at both timepoints, with the two mimics exerting a less marked influence compared with the acetone control. By contrast, the treatments had a lower impact on fungal communities with a less clear separation between conditions and a non-significant effect on β-diversity (PERMANOVA, *P* > 0.05) (Fig. 1D).

Since all the factors as well as their interactions showed a high impact on prokaryotic community assembly, PERMANOVA was performed testing of the impact of treatments at individual time points/compartments combinations (Table S3). Treatments had a significant impact on bacterial communities at T1 in both root endosphere and unplanted soil but not on rhizosphere. At T2, treatments significantly influenced the assembly of prokaryotic communities in all rhizo-compartments with the highest effect on the root endosphere (29.36% of explained variance). Still, no treatment effect was detected in fungal communities. In pairwise PERMANOVA analysis (Table S4), it was evident that the individual contribution of the different molecules influences the prokaryotic community abundance: the influence of zaxinone and MiZax5 treatments was significant in the unplanted soil, compared to the control, and in endosphere, especially at T2, all the tested molecules gave a significant impact.

Such variations in community assembly were evident when comparing relative abundances of the most abundant bacterial orders. For example, zaxinone treatment decreased the relative abundance of Pedospherales in the endosphere, while MiZax3 promoted their abundance in the rhizosphere at T1. Both MiZax molecules significantly decreased the amount of Polyangiales in the soil at T1 while at T2 MiZax5 decreased Rhizobiales levels. Besides, at T2, zaxinone significantly lowered the relative abundance of Anaerolinales in soil, while MiZax3 increased Chitinophagales in the endosphere (Figure S4 A-B). As reflected in previous PERMANOVA analysis, minor variations occurred in mycobiota at T1: at this time point, MiZax5 led to significantly higher Pleosporales levels in the root endosphere (Figure S5 A-B).

No effect of the treatments was observed on bacterial and fungal Shannon index (*α*- diversity) (Fig. 1E and Figure S6) at both time points for both soil and rhizosphere samples. A discernible trend toward an increase in diversity was evident in the root endosphere during the reproductive stage (T2) of the 16S rDNA amplicon dataset irrespective of the treatments (Figure S6C).

Furthermore, looking at the Shannon diversity index, differences between T1 and T2 within each treatment emerged indicating that zaxinone and MiZax can influence microbiota dynamics across plant phenological stages (Fig. 1E-F and S6). In more detail, in the endosphere the prokaryotic -diversity increased across time points for all the treatments considered, including the acetone control (Fig. 1E and S6). Regarding the fungal communities, zaxinone and MiZax5 elicited a substantial elevation in -diversity from T1 to T2 (Fig. 1F) while no effect emerged in the control. This trend is also detectable in the rhizosphere, where zaxinone increased fungal -diversity across time points (Figure S6B).

Notably, no discernible alterations in *α*-diversity were noted in the unplanted soil samples among T1 and T2, when compared with the trend of the acetone control, except for an increase in MiZax5 treatment for the prokaryotic community (Figure S6A). Overall, these results indicate that zaxinone and MiZax treatments exerted a mild effect on the prokaryotic community assembly with variations mainly related to compartments and time points while having a minor impact on the fungal community. Nevertheless, when comparing time points within the same treatment, it was found that zaxinone increased fungal *α*-diversity in root endosphere and rhizosphere in T2 *vs.* T1 compared to the control acetone condition, while MiZax5 had the same effect in unplanted soil considering the prokaryotic communities. This highlights the possible role of Zaxinone and its mimics in shaping root and soil communities across different plant life stages.

### Zaxinone and MiZax modulate microbial recruitment dynamics along the soil-root interface

**Fig. 2:**
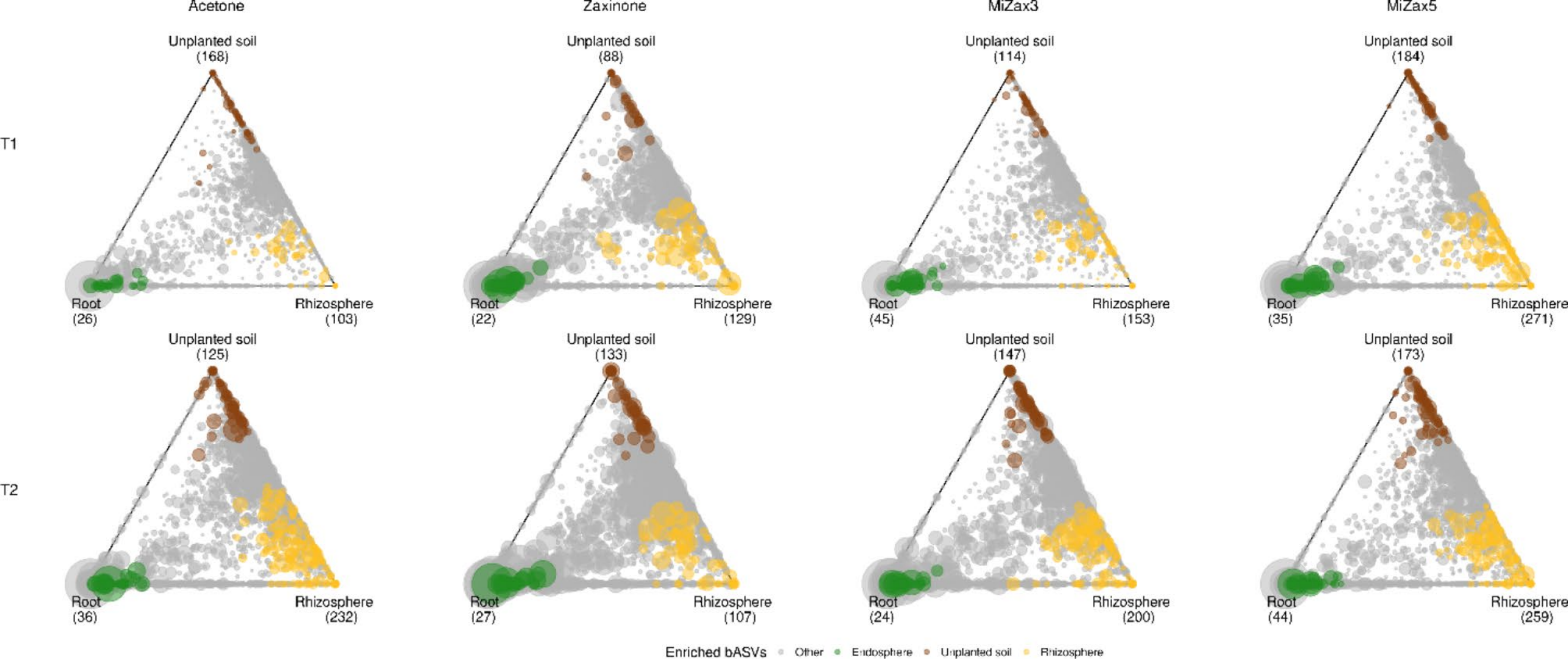
Ternary plots highlighting the distribution of the most abundant bacterial ASVs in the three root compartments for each hormonal treatment and timepoint. Each ASV is represented by a circle whose size is proportional to the average relative abundance across the three compartments. ASVs enriched in each specific compartment (significantly most abundant compared to the two others) are coloured (brown in unplanted soil, yellow in rhizosphere and green in root endosphere) while those not significantly enriched in any compartments are indicated in gray. The exact number of compartment-enriched ASVs is indicated in brackets at the triangle vertices. Only the most abundant ASVs that passed the HTS-filtering step prior to differential abundance analysis with DESeq2 (FDR < 0.05) were plotted here. Each plot column indicates a different treatment while row depicts ternary plot for each timepoint as indicated in the figure text annotation.

To investigate the recruitment of microbiota by rice plants along the soil-rhizosphere-endosphere continuum and to assess the potential interference of zaxinone and MiZax treatments in this process, compartment-specific ASVs, defined as those occurring in higher abundance in a particular compartment compared to others across treatments and time points were analyzed. The results revealed distinct patterns of bacterial taxa enrichment across compartments in the various treatments, exhibiting a pronounced timepoint-dependent trend. Specifically, at T1, there was an increase in the number of rhizosphere-enriched taxa under zaxinone, MiZax3, and MiZax5 treatments (Fig. 2). Concurrently, there was an increase in root endosphere-enriched taxa compared to the acetone control in all treatments except for the zaxinone treatment. At T2, a decrease in rhizosphere- and root-enriched ASVs upon the treatment with zaxinone and MiZax3 was observed, whereas the application of MiZax5 caused an increase in root- and rhizosphere-enriched taxa (Fig. 2). Overall, MiZax5 treatment proved most effective in increasing the number of highly specific rhizosphere taxa at both time points.

Notably, treatments had also an effect on soil-enriched ASVs. With the exception of MiZax5, all the treatments reduced and increased soil-enriched ASVs at T1 and T2, respectively (Fig. 2).

At each time point, all treatments showed a shared core of root-enriched taxa which included Comamonadaceae, Rhizobiaceae, Chloroflexaceae, and Microscillaceae at T1 with the addition of a *Novosphingobium* sp. at T2. Among this root-specific set of taxa, MiZax treatments consistently recruited specific *Acidibacter* sp., *Devosia* sp., and Comamonadaceae ASVs at T1 (Figure S7).

Altogether, these data suggest that zaxinone and MiZax treatments exert different effects on microbiota recruitment according to the timepoint considered. Remarkably, during the 15 days following the application of treatments (T1 sampling), plants exhibited the recruitment of distinct endosphere and rhizosphere communities. Nevertheless, by T2, the bacterial community assemblies in both compartments displayed increased homogeneity, marked by a reduction in compartment-specific taxa and an augmentation of taxa shared among different compartments. The sole exception to this trend was observed in MiZax5, which consistently maintained highly compartment-exclusive communities at both time points and across compartments (Fig. 2 and S8).

### Zaxinone and MiZax modulate bacterial and fungal taxa in the root endosphere

**Fig. 3:**
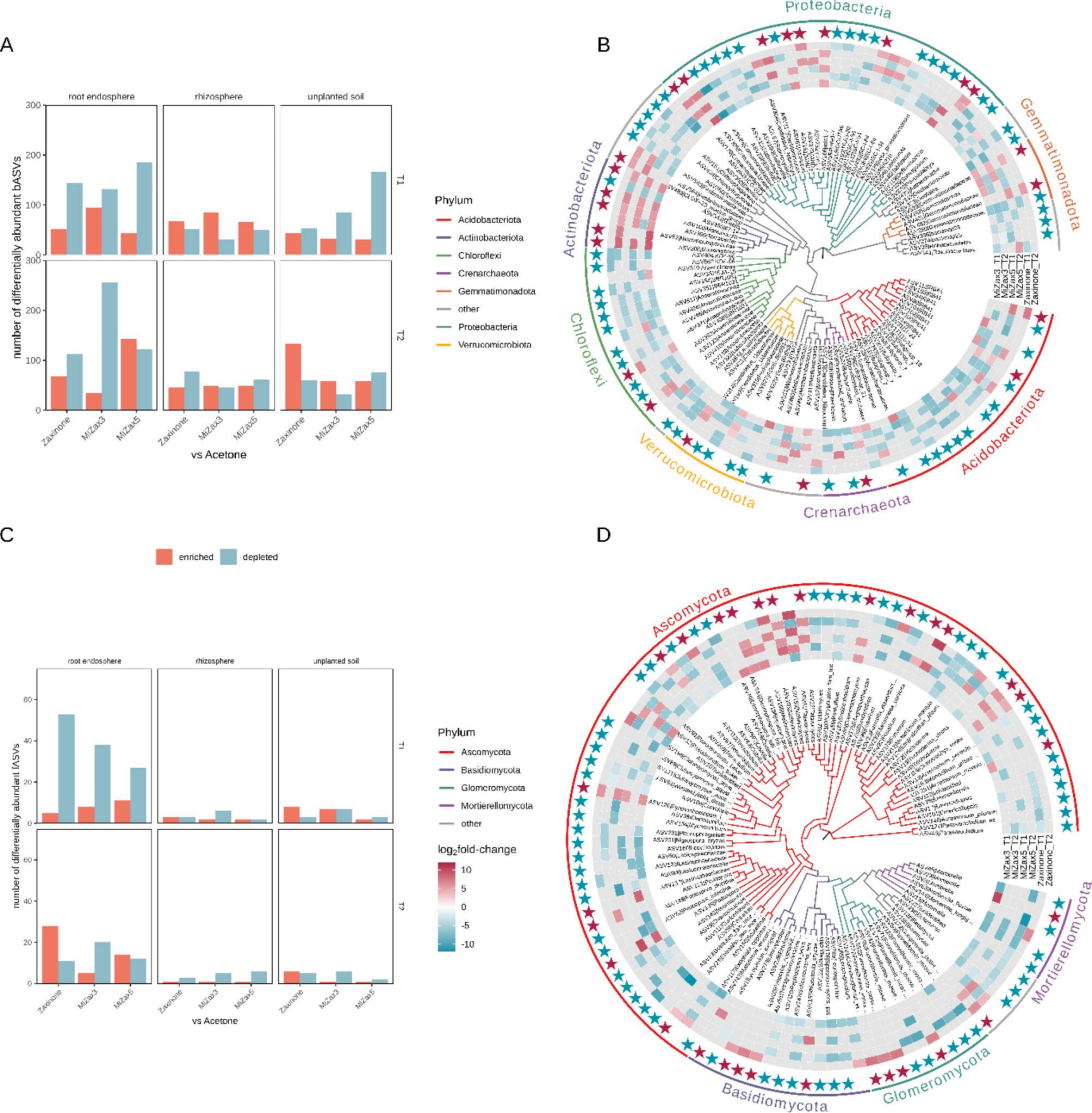
Differential abundance of prokaryotes and fungi in zaxinone and MiZax treatments compared to the control (acetone). (**A**-**C**) Number of enriched and depleted ASVs in the three compartments considered (endosphere, rhizosphere, unplanted soil) at tillering (T1) and milky-stage maturation (T2) in prokaryotes (A) and fungi (C). (**B**-**D**) Circular phylogenetic tree and heatmap of the bacterial (C) and fungal (D) ASVs with higher or lower microbial loads in the endosphere of zaxinone/MiZax treated plants compared with acetone-treated controls. Only ASV with a relative abundance higher than 5% and classified at least at Family rank are displayed. Maximum likelihood (ML) phylogenetic trees were constructed using differentially abundant ASV sequences and tree branches were coloured according to taxonomy (*sensu* UNITE v8) at the phylum level. The heatmaps shows log_2_fold-change values indicating ASVs enriched (red) and depleted (blue) in treatments compared with the acetone control. Stars represent ASVs consistently enriched or depleted compared to the control in at least two conditions.

The analysis of differential abundance (Fig. 3) at the ASV level revealed that zaxinone and MiZax molecules exerted the most substantial impacts on root endosphere communities, evidenced by a higher number of differentially abundant taxa at both T1 and T2, for both prokaryotic and fungal communities. In contrast, the rhizosphere and soil exhibited a lower number of taxa with altered abundance following treatments. This pattern was notably pronounced in prokaryotic communities (Fig. 3A), and a comparable trend was observed in Fungi, albeit with a limited number of differentially abundant taxa across conditions in the latter case (Fig. 3C). Furthermore, our observations indicate that at T1, the majority of taxa exhibited a negative response to almost all treatments in the root, rhizosphere, and soil. In the root endosphere, the number of enriched/depleted prokaryotic taxa at T2 diverged upon zaxinone and MiZax3 treatments. In particular, upon MiZax3 treatment an increase of depleted taxa was identified while the MiZax5 treatment resulted in a higher number of enriched taxa compared to the other treatments (Fig. 3A). Considering the taxonomic diversity of differentially abundant taxa in the root endosphere, it was observed that depleted taxa spanned across the bacterial phylogeny, with the exception of Actinobacteriota which mostly increased in their abundance in all treatments at T1 (Fig. 3B). The phyla Acidobacteriota and Proteobacteria are generally depleted. In particular, within Proteobacteria the family Nitrosomonadaceae (genus *Ellin6067*) is decreased in our dataset after the treatments at the first timepoint, while the relative abundance of Comamonadaceae (Proteobacteria) increased with MiZax5 treatment.

The application of the molecules was consistently linked to a reduction in Chloroflexi abundance both in the vegetative and in reproductive plant stages.

Among depleted taxa, Fimbriimonadaceae (Armatimonadota phylum) emerged especially upon zaxinone and MiZax5 treatments. This taxon is usually detected in ANAMMOX (ANaerobic AMMonium OXidation) consortia, implying that the Fimbriimonadaceae family either contains ammonia-oxidizing taxa or has positive interactions with ammonia-oxidizing bacteria, favoring the ammonia-oxidizing processes^86^.

Additionally, a diminished abundance of sulfate-reducing bacteria, such as Geobacteraceae, was observed across all treatments at T2.

Notably, treatments significantly influenced Archaeal taxa, with methanogens (primarily *Methanosarcina*, *Methanobacterium*, *Methanosaeta*, and *Methanocella* genera) generally exhibiting increased abundance in treated samples compared to the control, particularly at T1.

Notably, an increase in the abundance of Actinobacteriota, particularly at T1 was detected. This group encompasses taxa well-known to establish beneficial interactions with plants, acting both in the rhizosphere and as endophytes, stimulating plant growth and enhancing disease resistance^87^. Conversely, other groups well-acknowledged to include plant-beneficial species including Sphingomonadaceae (Proteobacteria) and Bacillaceae (Firmicutes), decreased in abundance suggesting a treatment-induced shift of these components.

Considering Fungi, our findings revealed no distinct phylogeny-related differences, as the taxa depleted in the root endosphere were distributed across all major phyla, with only a few exceptions. In particular, almost all the ASVs belonging to Helotiales (namely *Talaromyces*, *Dimorphospora*, *Meliniomyces*, and *Hyaloscypha* ASV) were enriched across treatments (Fig. 3D). This group includes soil fungi with marked organic matter degradation abilities that are known to associate with plant roots as endophytes and symbionts^88^. Within Mortierellomycota, *Glomeromycotina * formerly Glomeromycota, genera such as *Funneliformis * and *Claroideoglomus*-related ASVs in Fig. 3D*)* and *Mortierellomycotina* (formerly Mortierellomycota), seem to be mostly depleted in this compartment, with the exception of zaxinone- and MiZax5-treated plants at T2, which show an increment of different taxa, especially the genera *Funneliformis* and *Mortierella*. However, these changes do not reflect any significant changes in the whole AMF community at higher taxonomic level such as families, which is mainly composed of Glomeraceae, Paraglomeraceae, and Claroideoglomeraceae at variable abundances according to the phenological stage and additionally being slightly affected by treatment (Figure S8). Lastly, two ASVs pointing to the *Trichoderma *genus showed an increase in its abundance at T2 upon both Zaxinone and Mizax5 treatment. This fungus is known to exert plant-beneficial abilities, being particularly active as a biocontrol agent and increasing yield in rice^89^.

Taken together, these data suggest an overall impact of zaxinone and its mimicking molecules on the abundances of numerous microbial groups, which turned out to be mostly decreased at the first time point, with some exceptions. At T2, the influence of the treatments seems to be globally less pronounced.

### Zaxinone and MiZax treatment impact microbiota network dynamics more at the vegetative than at the reproductive stage

**Fig. 4:**
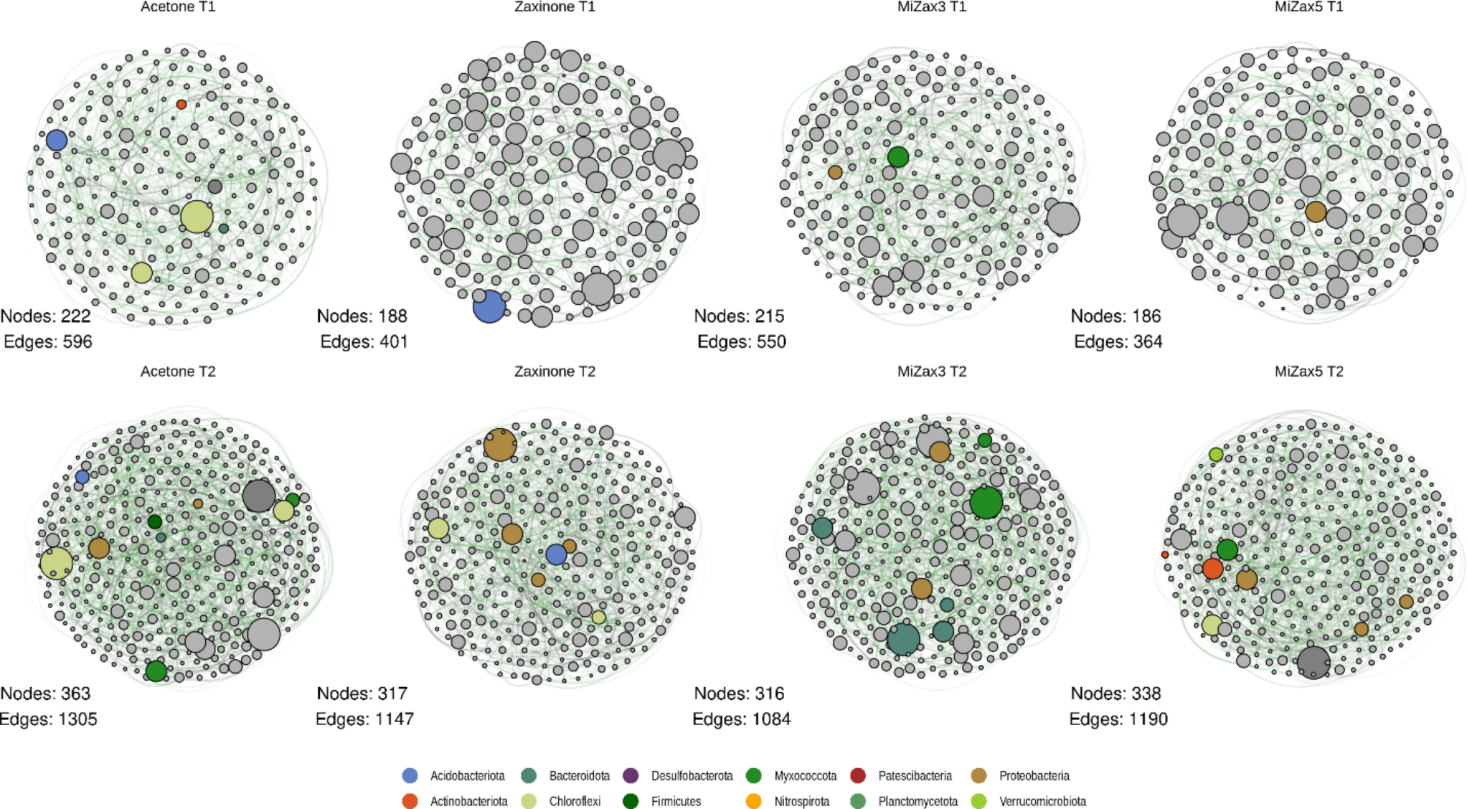
Co-occurrence networks displaying interactions between bacterial ASVs in the rice root endosphere at each treatment and timepoints. Different treatments are displayed in columns while timepoints in rows. Microbial hubs (see methods) are coloured according to their taxonomy, at phylum level; the node size is proportional to degree, while edge transparency indicates the correlation strength. The number of nodes and edges for each graph is indicated and the Fruchterman-Reingold layout has been used.

To track for changes in co-occurrence dynamics determined by treatments on the bacterial endosphere community and to identify hubs-taxa (i.e. ’keystone microbes which drive the structure of the community), co-occurrence networks were constructed using Sparse Inverse Covariance estimation (SPIEC-EASI) for each timepoint and treatment considered (Fig. 4). Globally, structures of the community in each treatment at each timepoint showed comparable network-level metrics, with a higher ratio of positive correlations between taxa (mean 61.1% positive edges) and an overall similar modularity, centrality metrics, and cohesion (Table S6). Notably, at the milky-stage (T2), communities showed increased connectivity, nodes degree, and number of hubs-taxa identified, indicating a higher community complexity.

However, a treatment-dependent modulation was observed in most of the node-level metrics analyzed (Figure S9). At T1 all the treatments significantly decreased the degree distribution (number of connections,* i.e*. edges, established by each node). Zaxinone and MiZax5 treatments significantly increased betweenness *i.e*. the extent to which a node lies in the shortest path connecting other nodes and decreased closeness centrality *i.e*. the average distance to all other nodes. Further, the eigenvector-centrality and the HUB score, which both indicate the amount of connections towards highly influential taxa, were positively impacted by the treatments with the exception of MiZax5. In addition, in both zaxinone and MiZax5 the betweenness centrality significantly increased while closeness centrality decreased (Figure S9). Altogether, these metrics indicate that treatments increased distance between taxa and increased the occurrences of more isolated sub-communities, in terms of connections while decreasing the overall connections between taxa (lower degree). At T2, the degree distribution became more uniform across treatments, with the exception of MiZax3, where significantly fewer connections emerged. Concurrently, at the same timepoint, all treatments led to an increase in node closeness, and, except for MiZax5, also in betweenness centrality.

Interestingly, by comparing the inferred network metrics with those of randomly generated graphs (see methods; Table S6), it was found that at both time points network structures were not casual, highlighting that reconstructed community dynamics were meaningful.

**Fig. 5:**
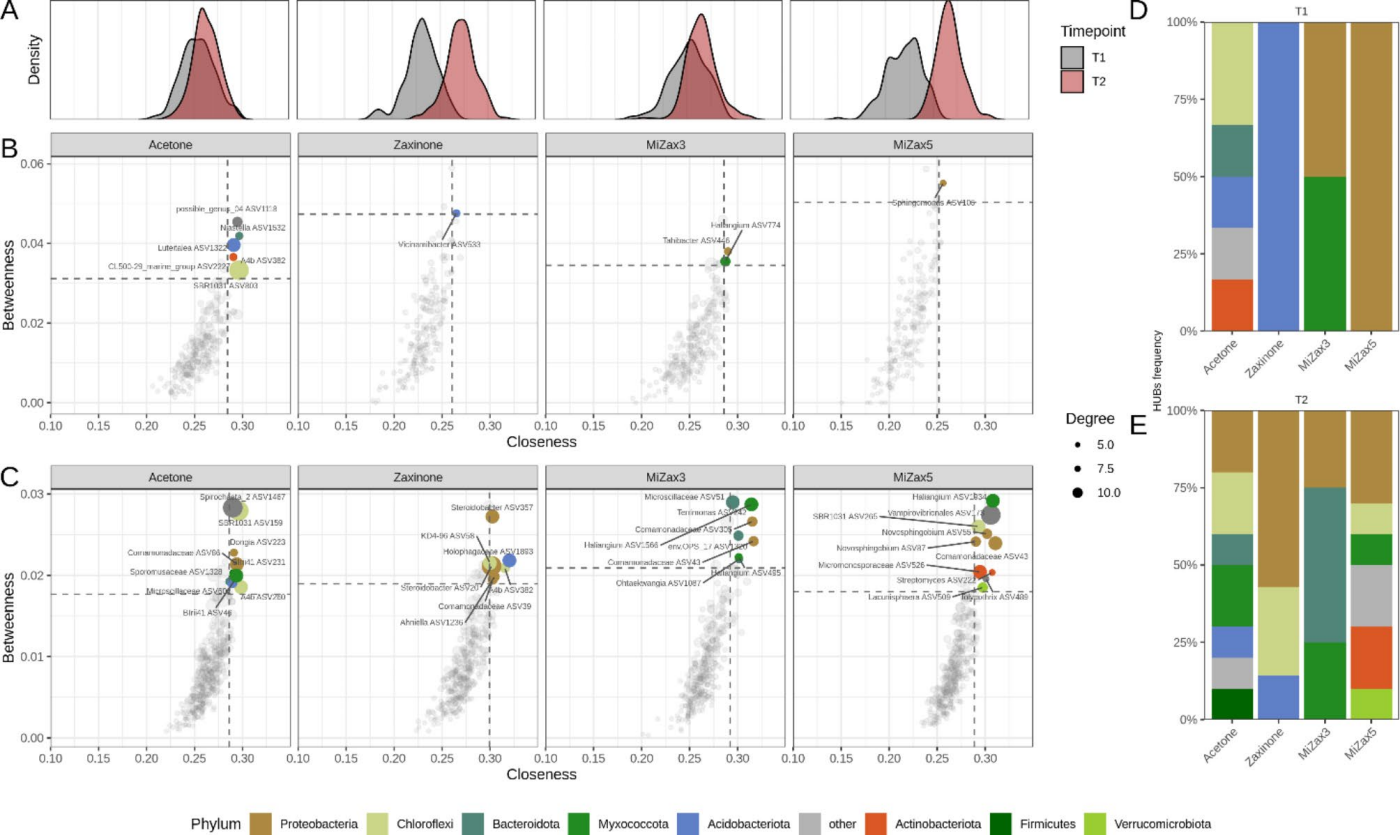
Analysis of bacterial hubs in the endosphere of zaxinone and MiZax-treated rice roots. (**A-C**) Identification of microbial hubs within *Oryza sativa* root endosphere microbiota. Co-abundance networks inferred with SPIEC-EASI were used to identify microbial hubs defined as the nodes with betweenness centrality (i.e. the fraction of shortest paths passing through the given node) and closeness-centrality (i.e. the average shortest distance from the given node to other nodes) metrics above the top 5% values in the distribution (dotted lines in B and C). Hubs are coloured according to Phylum and dot size is proportional to node’s degree (exponential scale). Density plots (A) display the distribution of the closeness-centrality values of ASVs (network nodes) across treatments (panels) and timepoints (color). (**D-E**) Frequency of phyla by treatments and time-points in the microbial hubs identified in the network analysis.

To offer a more detailed insight into the impact of treatments on the community network structure, hub-taxa, *i.e.* the nodes characterized by higher closeness and betweenness centrality (top 5% of the distribution, Fig. 5) were identified. At T1 most of the hubs-taxa detected across treatments belong to Proteobacteria, Myxococcota, and Acidobacteria including *Sphingomonas*, *Haliangium*, *Vicinamibacter*, and *Tahibacter* genera. Most of them were already known as keystone species in root- or plant-associated communities and hold plant-growth-promoting capacities. The analysis indicated that treatments resulted in a reduction of hub-taxa at T1 (from 6 to 1–2 hub-taxa in the control and treatments, respectively), while minor to no differences were detected at T2. In the control (acetone), hub-taxa primarily consisted of Chloroflexi (A4b family), Acidobacteriota, Firmicutes, Actinobacteriota, and Bacteroidota members, with the inclusion of Proteobacteria (Comamonadaceae) at T2. Moreover, in the examination of the node’s closeness distribution, a notable shift towards lower values was observed in zaxinone and MiZax5 at T1, reflecting the reduced number of established edges (degree metrics, Fig. 5A).

At T2, keystone taxa increased compared to T1 in all treatments. Several Comamonadaceae ASVs (proteobacteria), acknowledged for their prevalence in rice-associated root communities^84^, were identified as keystone taxa in all treatments as well as in the control. Under MiZax5 treatment, *Novosphingobium* and *Streptomyces*, two well-acknowledged PGP species, emerged as hub-taxa. Interestingly, in both MiZax3 and MiZax5 treatments, *Haliangium* occurred as a keystone species.

Overall, analysis of co-occurrences indicated that all the treatments decreased the overall network complexity promoting the isolation of sub-communities and decreasing the number of hubs at the vegetative stage (T1), while at the reproductive stage (T2), in treated plants and in particular upon MiZax5 application, significant interactions between taxa were re-established in a similar manner to the acetone control, though with an array of hub-taxa that seems to be specific for each condition considered.

### Zaxinone and MiZax treatment induce alterations in plant’s primary metabolism and grain nutrient content

**Fig. 6:**
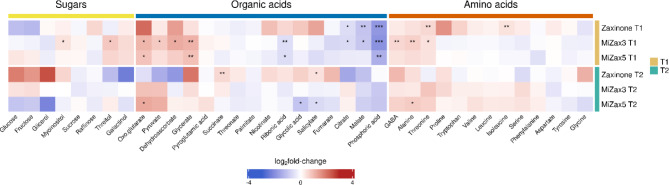
Profiling of primary metabolites of rice shoots treated with zaxinone and MiZax using targeted GC-MS. Heatmap plot showing log_2_fold-change values (treatments *vs.* acetone control) of metabolites detected. Blue and red colors depict a decrease and increase in metabolite levels respectively. Asterisks indicate significantly-supported differences between each metabolite levels in treated *versus* acetone control condition according to Dunn’s post-hoc test after Kruskall-Wallis (* *P* < 0.05, ** *P* < 0.01, *** *P* < 0.001).

At the metabolomic level, the effects of zaxinone-related compounds and the associated root microbiota composition on treated and non-treated rice plants were determined by collecting shoots from the same rice plants used for the metabarcoding analysis at T1 and T2. Through targeted GC-MS analysis, approximately 40 primary metabolites, including amino acids, organic acids, and sugars, were identified as differentially accumulated metabolites (Fig. 6). Noteworthy alterations in metabolite levels were observed across various metabolite classes in green tissues. During the vegetative stage (T1), MiZax3 and MiZax5 treatments induce a trend of increase in the sugar content. In particular, threitol and myoinositol are significantly more abundant in the shoots upon MiZax3 treatment. Conversely, zaxinone exhibited a contrasting trend in sugar content at T1 compared to its synthetic mimics. As the plants progressed to the reproductive stage (T2), a higher trend in sugar content (glucose, glycerol, myoinositol, and fructose) has been reported under zaxinone treatment, whereas the same compounds appeared rather reduced following MiZax5 treatments, though none of the detected changes turned out to be statistically significant. A general accumulation of amino acids was observed upon treatments: in particular, an increase in the levels of different free amino acids (alanine, threonine, and GABA) was observed at T1 upon MiZax treatment. At the same timepoint, zaxinone treatment induced an accumulation of isoleucine, proline, and threonine while at T2 an increase in alanine was observed upon MiZax5. While the effect of the treatments on sugar and amino acid accumulation seems to be limited, the organic acid pattern turned out to be more influenced, especially at T1. At that time point, MiZax3 induced TCA cycle intermediates, including glycerate, pyruvate, and (2)-oxoglutarate. Similarly, at both time points MiZax5 increased glycerate and (2)-oxoglutarate content. While at T1 malate and citrate showed a statistically-supported lower abundance in shoot treated with MiZax3 and zaxinone, a slight decrease in shoot treated with MiZax5 was observed. Ribonic acid was also negatively affected mainly by both MiZax. A substantial reduction in phosphoric acid levels in the shoots after all zaxinone-related compounds treatments was also observed at T1. By contrast, an increase in salicylate levels in the shoots was observed at T2 following zaxinone treatment, whereas MiZax3 and MiZax5 exhibited an opposite trend.

To a certain extent, the metabolomic profiles are in agreement with the results obtained by Wang et al.^35^ , which reported an increased concentration of free amino acids and succinate in the shoot of rice plants treated with zaxinone. However, there are some discrepancies in other metabolic pathways, namely sugar metabolism and organic acids. This may be ascribed to differences in the experimental set-up (*i.e.* plant’s phenological stage and compound applications in hydroponic or soil system). Furthermore, our findings indicate that MiZax3 is the most effective compound in modulating the plant metabolome, particularly at T1.

In order to determine the impact of zaxinone and MiZax compounds and the relative root-associated microbiota community on seed nutrient content, the starch content, the antioxidant capacity, and the mineral nutrient profile were evaluated.

While the antioxidant activity and the starch content did not change across treatments, some differences in grain mineral nutrition were observed. Indeed, a higher content of zinc (33.87 ppm) and copper (5.73 ppm) in seeds of plants treated with zaxinone compared to control acetone plants was detected. Conversely, a decrease in manganese content was observed in seeds of plants treated with both MiZax3 and MiZax5 (Figure S10).

## Discussion

### The microbiota analysis reveals a community typical of paddy soils

Rice fields represent a peculiar environment for the microbial soil communities. Due to the periodic flooding, this habitat is characterized by oxygen-limited conditions that shape the microbiota assembly. Bacterial communities typically include both aerobic and anaerobic taxa^90^. The overall microbiota assembly from unplanted soil revealed a prokaryotic composition typical of paddy environments, including Gemmatimonadetes, Chloroflexi, Acidobacteria and Actinobacteria, and the archaeal phylum Crenarchaeota^90–92^. On the fungal side, our analysis revealed a relevant proportion of Leotiomycetes in all the conditions considered. This group of fungi includes good organic matter decomposers that can tolerate high levels of heavy metal contaminants usually found in paddy soils^93^, and can associate with plant roots living as endophytes.

### Impact of zaxinone and its mimics on rhizomicrobiome diversity and composition

So far, the effects of exogenous treatment with zaxinone and its mimics (MiZax3 and MiZax5) were investigated considering the growth promotion activity, the regulation of SLs biosynthesis, and the AM symbiosis^32,36^. Here, we provide comprehensively new information about the impact of these compounds on paddy soil and rice root-associated microbes and how these effects systemically influence rice metabolomic and grain nutrient profiles considering different developmental stages.

The metabarcoding analysis highlighted that rice microbiome assembly is mainly influenced by compartment niche and developmental stage as already reported for rice and other plants^94–96^, regardless of zaxinone and MiZax treatments. In contrast to the findings reported by Zangh and colleagues^95^, which observed a reduction in root microbial community richness in the later stage of the rice life cycle, our data reveals a general increase in *α*-diversity at T2 compared to T1 across the three considered compartments (unplanted soil, rhizosphere, and endosphere), regardless of the treatments. This increment was evident in the endosphere particularly for the prokaryotic community while the *α*-diversity of the fungal community increased over time only under zaxinone and MiZax5 treatments.

Notwithstanding the compartment and developmental stage, zaxinone and MiZax treatments significantly influenced the *β*-diversity of prokaryotic microbial communities. Notably, we highlighted a shared pattern between MiZax3 and MiZax5 more similar to the acetone control, whereas a clear separation was evident for samples treated with zaxinone.

Studies focused on plant traits demonstrated that MiZax3 and MiZax5 exhibit zaxinone-like activity by rescuing the root growth of a zaxinone-deficient rice mutant. They also promote overall growth and decrease SLs content in both roots and root exudates of wild-type plants^36^. However, contrasting results were observed when mycorrhization was considered. Specifically, the application of 5 µM MiZax did not negatively impact AM fungal root colonization, whereas zaxinone treatment markedly reduced AM mycorrhization^32,36^. These findings suggested that zaxinone and MiZax compounds can be interchanged when plant traits are concerned but more caution is needed considering plant-microbe interactions. Our metabarcoding data are consistent with these observations. As shown by the analysis of compartment-specific taxa, the different pattern between MiZax and zaxinone treatment seems to be more evident at T1. Herein, a stronger polarization between root and rhizosphere prokaryotic communities occurs in MiZax3 and MiZax5 compared to the acetone control. Conversely, in the zaxinone treatment, a higher overlap between these two compartments is detected at both developmental stages.

In particular, in the endosphere at T1, we identified an increased number of ASVs which are key soil-beneficial bacterial taxa, such as Comamonadaceae, *Acidibacter*, and *Devosia*, enriched under MiZax3 and MiZax5 treatments. Acidibacter and Comamonadaceae have normally high phosphorus solubilizing activity^97,98 ^and in addition, Comamonadaceae has also the ability to solubilize potassium, zinc, and nitrogen and its abundance has been related to disease-suppressive soil^98,99^. *Devosia *has been reported as nitrogen-fixing bacteria which also alleviate abiotic stress^100 ^and recently has been reported to be enriched on AM extraradical hyphae and mycorrhizal root^101^. Furthermore, two novel *Devosia *species recently isolated from the rice rhizosphere have been demonstrated to produce IAA and siderophores^101^.

Further, in the rhizosphere MiZax3 and MiZax5 shared the accumulation of keystone rhizobacteria taxa such as Pedosphaeraceae^102^, and *Nitrosarchaeum *which is predicted to be an anaerobic hydrocarbon-degrading bacteria in the subsurface soil^103^. By contrast, at T2 in both zaxinone and MiZax3, rhizosphere and endosphere prokaryotic communities were more similar, while MiZax5 still maintained the pattern shown at T1.

By contrast, the impact of the treatments on soil communities was detectable but negligible in term of the amount of regulated taxa. It is plausible to hypothesize that the soil environment more effectively buffers exogenous environmental changes, potentially due to its higher microbial abundance compared to root tissues. As an alternative, we can hypothesize that the applied molecules only exert a limited direct effect on the microbial communities, such an outcome being magnified by the plant. Zaxinone and its mimics are in fact perceived by the plant, which might undertake modification of its hormonal balance and/or rhizodeposition that could result in a differential recruitment of endosphere/rhizosphere microbiota.

Coupled with the evidence that the application of MiZax3 and MiZax5 differentially modulated the abundances of bacterial ASVs both in the endosphere and in the rhizosphere, this data suggests that each molecule has a specific impact on shaping the bacterial community. Compared with the acetone control, at T1, all the molecules exerted the most significant impact on the root endosphere bacterial and fungal communities with a widespread depletion of taxa, while an opposite pattern was observed in the rhizosphere where the compounds supply incremented the number of enriched taxa, at least for prokaryotic communities. At T2 a higher ratio of depleted prokaryotic taxa was observed in the endosphere following treatment with zaxinone and MiZax3 while MiZax5 has a milder effect, with enriched taxa prevailing on the depleted ones. The endosphere mycobiota was slightly less impacted by treatments compared to T1. In the rhizosphere and in the unplanted soil, all the treatments displayed a limited impact in terms of enriched/depleted taxa, irrespective of the timepoint. These findings suggest that zaxinone and MiZax treatments exert the highest impact on microbial communities in the root endosphere, suggesting once again that these dynamics are likely influenced by plant-mediated processes.

In the analysis of the taxonomic diversity of differentially abundant taxa in the root endosphere, it was observed that depleted taxa were widely distributed across the bacterial phylogeny. Notably, Actinobacteriota was an exception, mostly showing an increase in their abundance in all treatments at T1. This phylum is recognized as the producer of many bioactive metabolites in agriculture, such as insecticides, herbicides, fungicides, and growth-promoting substances for plants^104^. By contrast, the abundance of Acidobacteria decreased drastically, overall. Interestingly, a lower abundance of Acidobacteria was also found in rice *d17 *mutant lines defective for SLs biosynthesis^26^, suggesting that the decreased Acidobacteria abundance in the endosphere of zaxinone and MiZax treated plants could be dependent on the negative impact of these compounds on SLs biosynthesis.

At the same time, we observed an enrichment of Chitinophagaceae upon MiZax3 treatment. Since Chitinophagaceae was negatively associated with orobanchol^30^, their higher abundance could be related to the negative impact of MiZax on SLs biosynthesis.

Regarding the fungal community, we observed a depletion of Mucoromycota phylum. It is worth noting that different *Funneliformis *ASVs belonging to Glomeromycotina subphylum were depleted by zaxinone treatment at T1 while enriched at T2. By contrast, MiZax3 and MiZax5 have a mild impact at both time points. These findings are in line with our previous reports which displayed at early time points a strong reduction of AM symbiosis colonization in rice root treated with zaxinone while no negative effects were reported upon MiZax treatment^32,36^. The decrease of these taxa at a very early sampling time may be attributed to the lower SL content in root exudates induced by zaxinone treatment^33^. The SLs reduction potentially delays the mycorrhization process, which however was fully recovered over time.

The analysis of prokaryotic community interactions in the endosphere, revealed in zaxinone and MiZax-treated rice roots at T1 a decrease in network complexity and in a number of keystone taxa compared to the control, with the most pronounced effects for zaxinone and MiZax5 treatments, although few but significant plant-beneficial microbes emerged as hubs-taxa. However, at T1 all the network metrics indicated for most of the treatments a decentralization of microbe-microbe interactions compared to the control, with few but wider connections spanning through the network and possibly resulting in a community less dependent on a few hub species. These features suggest that at T1 the community may be more resilient to external perturbations, but further experiments are needed to confirm these speculations. At T2, in all treated plants and in particular, upon MiZax5, significant interactions between taxa were re-established in a similar way to the acetone control, with a shift in microbes potentially holding plant-beneficial traits among the identified keystone taxa.

Overall, our results indicate that all the treatments, to different extents, induce significant changes in root-associated rice microbial communities particularly at T1, with a stronger effect on prokaryotes. Notably, our data indicate that these changes modify the community network structure involving keystone taxa. However, at T2 the microbial communities dynamics were re-established suggesting a temporary delay in the microbial interaction established by treated plants compared to the control ones. Despite these changes, under most of the tested conditions, the hub taxa still include genera with well-acknowledged plant-beneficial traits such as *Sphingomonas*, *Streptomyces*, and *Haliangium*. This last species has been already recognized as a hub taxon in rice paddies, being very sensitive to external solicitations such as abiotic stresses (negative correlation) or biofertilization (positive correlation)^105^, and to be enriched in the rice root endosphere under the disturbance of barnyard grass (*Echinochloa crus-galli*^106^).

### Shoot metabolome and its correlation with the rhizomicrobiome

In the current study, we found that the soil treatment with zaxinone and its synthetic mimics systematically promote different metabolic pathways in the shoot of rice plants. Notwithstanding the treatment with zaxinone and MiZax exert comparable activity in the plant^36^, the impact on shoot metabolites and grain nutrient content display some differences that may be also attributed to the different rhizomicrobiota composition. It was determined in different host plants that different rhizomicrobiota communities play a significant role in metabolic profiles and mineral nutrient uptake at local and systemic level^107–109^.

The main metabolic differences in sugar, organic acid, and amino acid content have been detected at T1 upon MiZax3 treatment, while at T2 few differences have been observed between all treatments.

Wang et al.^35^ demonstrated that the growth-promoting effect of zaxinone is strongly linked with an increase in sugar metabolism in rice shoot at 24 h after treatment. In our conditions, differences in sugar content were not detected in the shoot of rice plants treated with zaxinone. In contrast, MiZax3 at T1 exhibited an increase in myoinositol and threitol content. Concerning the organic acids content, we observed at T1 an accumulation of oxoglutarate, pyruvate, dehydroascorbate, and glycerate in the shoot of MiZax3 treated plants, the content of some of them (oxoglutarate and glycerate) increased also upon MiZax5 treatment and as a general trend we observed an accumulation of these metabolites in shoots of plants treated with zaxinone. Interestingly, Wang et al.^35^ previously demonstrated the early accumulation of 2-oxoglutaric acid and glyceric acid in shoot as a response to zaxinone treatment, suggesting that also MiZax compounds play a role in organic acid metabolism. However, we measured a lower content of citrate and malate. Since both organic acids contribute to the acquisition of phosphorus in soils^110^, the reduced accumulation of these acids may be associated with the lower content of phosphoric acid observed in the shoots of all treated plants.

Concerning amino acid content, we identified an increment of GABA, alanine, and threonine content in both MiZax at T1. It is worth noting that upon these treatments at T1, we observed an increase of nitrogen-fixing bacteria such as Comamonadaceae and *Devosia*. Given their known ability to enhance plant nitrogen uptake and to produce amino acids, these bacteria may be accountable for the enhanced amino acid content in these plants^111^. In line with this hypothesis, Rahmoune and colleagues (2019) demonstrated that the inoculation of plant growth-promoting rhizobacteria (PGPR) in *Datura stramonium* affected significantly the amino acid content in both organs (root and shoot), highlighting a consistent increment of alanine in the shoot of inoculated plants.

### Impact of zaxinone and its mimics on grain nutrient content

Since it has been reported that zaxinone and MiZax also promoted the growth and yield of horticultural crops under open-field conditions^36–38^, we, therefore, investigated their impact on grain nutritional content. Regarding the mineral nutrients profile, we observed that zaxinone increased Zinc (Zn) and Cu content, while Mn was reduced under both MiZax treatments. Notably, elevated Zn concentration has been reported in rice grains of plants inoculated with β and ɣ-proteobacteria (i.e. *Sphingomonas* sp.,* Burkholderia cepacia*,* Pantoea rodasii*, and *Enterobacter* sp.) indicating a key role of rice-associated microbes in mineral grain content^112,113^.

Zinc deficiency is a major constraint to rice production and Zn is also often deficient in humans with rice-based diets^114^. The increase in Zn in the seeds of plants treated with zaxinone validates the use of this metabolite not only for improving rice growth but also for enhancing the nutritional aspect of the grain. Concerning Mn and Cu, both are considered essential elements for the growth and development of plants participating in many metabolic processes, including oxidation-reduction (redox), and photosynthesis^115^. The lower amount of Mn in grains of MiZax treated plants could be related to the increase in the level of chlorophyll and in the enhancement of photosynthetic activities reported in rice^35^ that could affect Mn homeostasis and translocation from shoot to the grains.

## Conclusion

Taken together, soil application of zaxinone and MiZax exerted a temporary strong effect on the endosphere microbial communities, particularly prokaryotes, while a minor impact on fungal communities was observed. Moreover, if the T1 showed a general depletion of the prokaryotes communities and a reduction of hub-taxa at T2 all treated plants, especially those treated with MiZax5, re-established significant interactions between taxa at a level comparable to the acetone control. This recovery can be due to the difference in the plant phenological stage or to enhanced resilience of the rhizomicrobiota community to tolerate exogenous treatments over time.

Among the communities impacted by treatments, a number of taxa potentially holding plant-beneficial traits were observed. However, since plant-growth promoting capacities cannot be confirmed by our metabarcoding approach, this evidence needs further validation by isolating strains or by in vitro tests.

Interestingly, the differences in microbial communities assembly highlighted at T1 among treatments and between the control are also partially mirrored in the metabolites profiles which displayed changes in sugar, organic acid, and amino acid content depending on the condition considered, while few differences between shoot of treated and control plants are observed at T2. Furthermore, the grain biochemical characterization revealed that these compounds are not only beneficial for increasing plant biomass and yield^36–38^, but also hold promise for enhancing the accumulation of zinc content in rice seeds.

With the adopted experimental set-up it was not possible to disentangle the effects of the treatments themselves on plant-associated communities from the changes arising from the different SLs exudation pattern exerted by MiZax application^33–35^. However, even to a much lower extent, treatments also impacted the soil prokaryotic community in absence of the plant suggesting a role for these molecules in shaping plant-associated microbial assemblages in a direct manner. This evidence opens new research questions, such as the understanding of contribution of the plant-mediated signaling on the changes observed here and whether these occur in a similar way across different crop plant models.

Overall, our results reinforce the practical use of zaxinone and MiZax application in the field as ecologically friendly biostimulants to enhance crop productivity without causing permanent disruption to the native rice root-associated microbiota, thereby paving the way for new strategies towards sustainable agriculture worldwide.

## Electronic supplementary material

Below is the link to the electronic supplementary material.

Supplementary Material 1

Supplementary Material 2

Supplementary Material 3

Supplementary Material 4

## Data Availability

Raw sequencing data have been submitted to the Sequence Read Archive (SRA) repository (NCBI) under accession No. PRJNA1060774. Additional data are publicly available at FigShare at https://doi.org/10.6084/m9.figshare.25586856.
