# Supplementary material for: Effect of exogenous treatment with zaxinone and its mimics on rice root microbiota across different growth stages
Source: Sci Rep. 2024 Dec 28;14:31374. doi: 10.1038/s41598-024-82833-6 (PMC11682185; doi:10.1038/s41598-024-82833-6)
Supplement: Supplementary file 1 — Supplementary Material 1 [file 41598_2024_82833_MOESM1_ESM.pdf]

Effect of exogenous treatment with zaxinone and its mimics on rice root microbiota across different growth stages.

Teresa Mazzarella[1§], Matteo Chialva[1§], Leonardo Perez de Souza[2], Jian You Wang[3], Cristina Votta[1], Rhowell Jr. Tiozon[4], Patrizia Vaccino[5], Alessandra Salvioli di Fossalunga[1], Nese Sreenivasulu[4], Tadao Asami[6], Alisdair R. Fernie[2], Salim Al-Babili[3*], Luisa Lanfranco[1], Valentina Fiorilli[1*]

[1] Department of Life Sciences and Systems Biology, University of Turin, Turin, Italy. [2]Max-Planck-Institute of Molecular Plant Physiology, Am Mühlenberg 1, 14476 Potsdam-Golm, Germany. [3]The BioActives Lab, Center for Desert Agriculture (CDA), Biological and Environment Science and Engineering (BESE), King Abdullah University of Science and Technology, Thuwal, 23955-6900 Saudi Arabia. [4]Rice Breeding Innovation Department, International Rice Research Institute, Los Baños, Philippines. [5]CREA-CI, Consiglio per la Ricerca in Agricoltura e l'Analisi dell' Economia Agraria, Centro di Ricerca Cerealicoltura e Colture Industriali, Vercelli, Italy. [6]Graduate School of Agricultural and Life Sciences, The University of Tokyo, 1-1-1 Yayoi, Bunkyo-ku, Tokyo 113-8657, Japan.

§These authors contributed equally to the work.

*Corresponding author. valentina.fiorilli@unito.it; salim.babili@kaust.edu.sa

Supporting Information

Supporting Figures

Figure S1. Rarefaction curves of prokaryotic (16S rDNA) amplicon libraries across compartments, treatments and time points.

Figure S2. Rarefaction curves of fungal (ITS2 rDNA) amplicon libraries across compartments, treatments and time points.

Figure S3. Unconstrained Principal coordinate analysis (PCoA) of Bray-Curtis dissimilarity of ASVs abundances across compartments, treatments and timepoints.

Figure S4. Relative abundance of selected bacterial orders in each treatment across timepoints and compartments.

Figure S5. Relative abundance of selected fungal orders in each treatment across timepoints and root compartment.

Figure S6. Shannon index values (α-diversity) grouped by compartment, treatment and timepoint in 16S and ITS2 amplicon libraries.

Figure S7. Heatmap showing the relative abundances of compartment-enriched bacterial ASVs and their overlap across treatments and timepoints.

Figure S8. Assembly and diversity of root-associated microbiota under zaxinone and MiZax treatments at tillering (30 days after transplanting, T1) and milky-stage maturation (90 days after transplanting, T2).

Figure S9. Node centrality metrics of the root endosphere bacterial co-occurrence networks for each treatment and timepoint (rows).

Figure S10. Analysis of biochemical parameters in rice grains from zaxinone and MiZax-treated plants.

Supporting Tables

Table S1. Soil physico-chemical characteristics of the native paddy soil used in the mesocosm experiment.

Table S2. PERMANOVA table (*adonis*, 9999 permutations) of the Bray-Curtis dissimilarities for bacterial and fungal microbial communities.

Table S3. PERMANOVA table (adonis, 9999 permutations) of the Bray-Curtis dissimilarities for bacterial and fungal microbial communities grouped by compartment and timepoint.

Table S4. Pairwise PERMANOVA table (*adonis*. 9999 permutations) of the Bray-Curtis dissimilarities for the different prokaryotes communities.

Table S5. Pairwise PERMANOVA table (*adonis*. 9999 permutations) of the Bray-Curtis dissimilarities for the different fungal communities.

Table S6. Network-level metrics for each of the bacterial community co-occurrences networks constructed.

Supporting Data

Dataset S1. Raw reads count and ASVs calling statistics for 16S and ITS2 rDNA amplicon libraries.

Dataset S2. Differentially abundant prokaryotic ASVs across treatments, timepoints and compartments.

Dataset S3. Differentially abundant fungal ASVs across treatments, timepoints and compartments.

Supplementary Figures

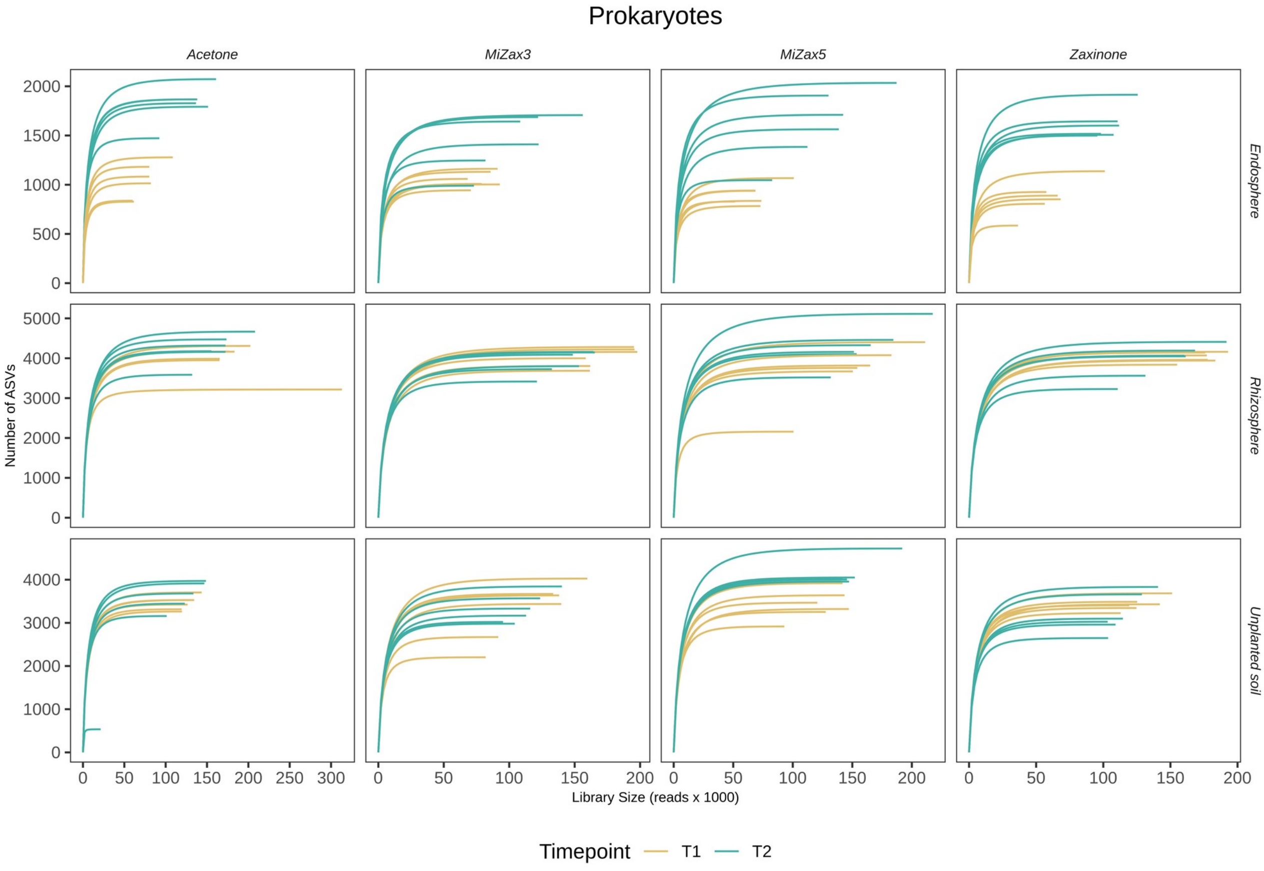

Figure S1. Rarefaction curves of prokaryotic (16S rDNA) amplicon libraries across compartments, treatments and time points. Timepoints are indicated with different colours; rows and columns indicate treatments and compartments, respectively.

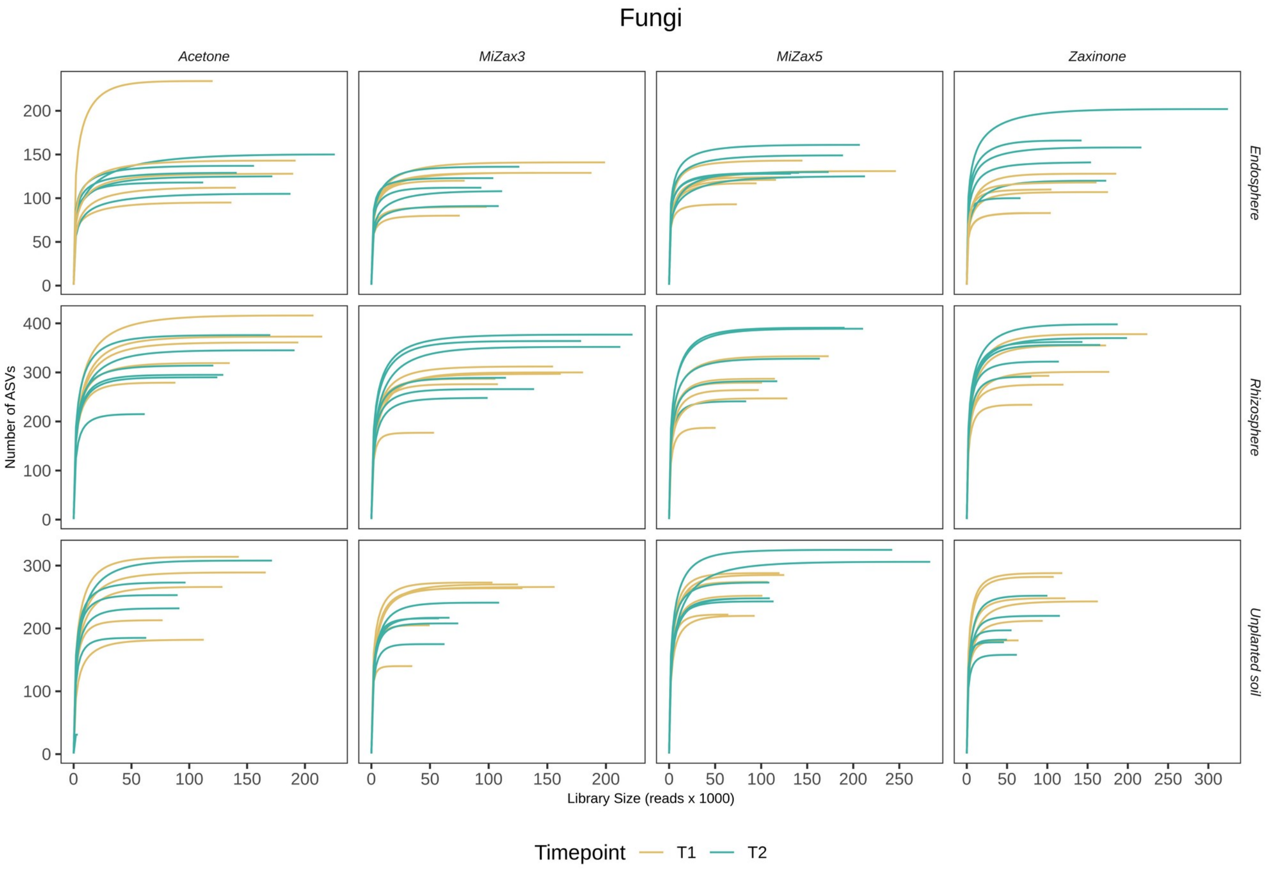

Figure S2. Rarefaction curves of fungal (ITS2 rDNA) amplicon libraries across compartments, treatments and time points. Timepoints are indicated with different colours; rows and columns indicate treatments and compartments, respectively.

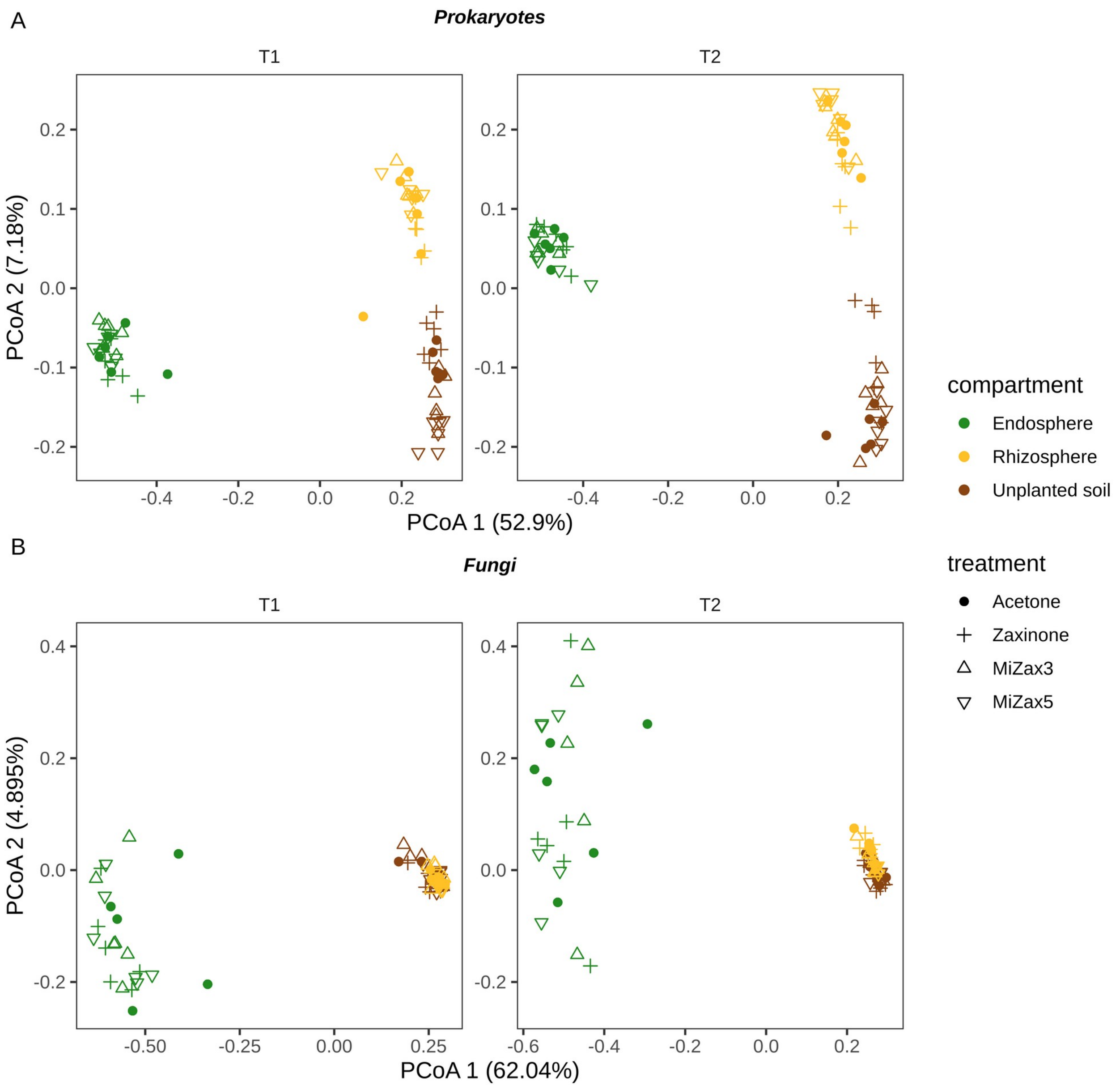

Figure S3. Unconstrained Principal coordinate analysis (PCoA) of Bray-Curtis dissimilarity of ASVs abundances across compartments, treatments and timepoints. PCoA for prokaryotic (**A**) and fungal (**B**) libraries. Each point represents a single library, treatments are indicated with different shapes while root compartments with different colours. T1, vegetative stage (60 days after transplanting); T2, milky stage maturation stage (120 days after transplanting).

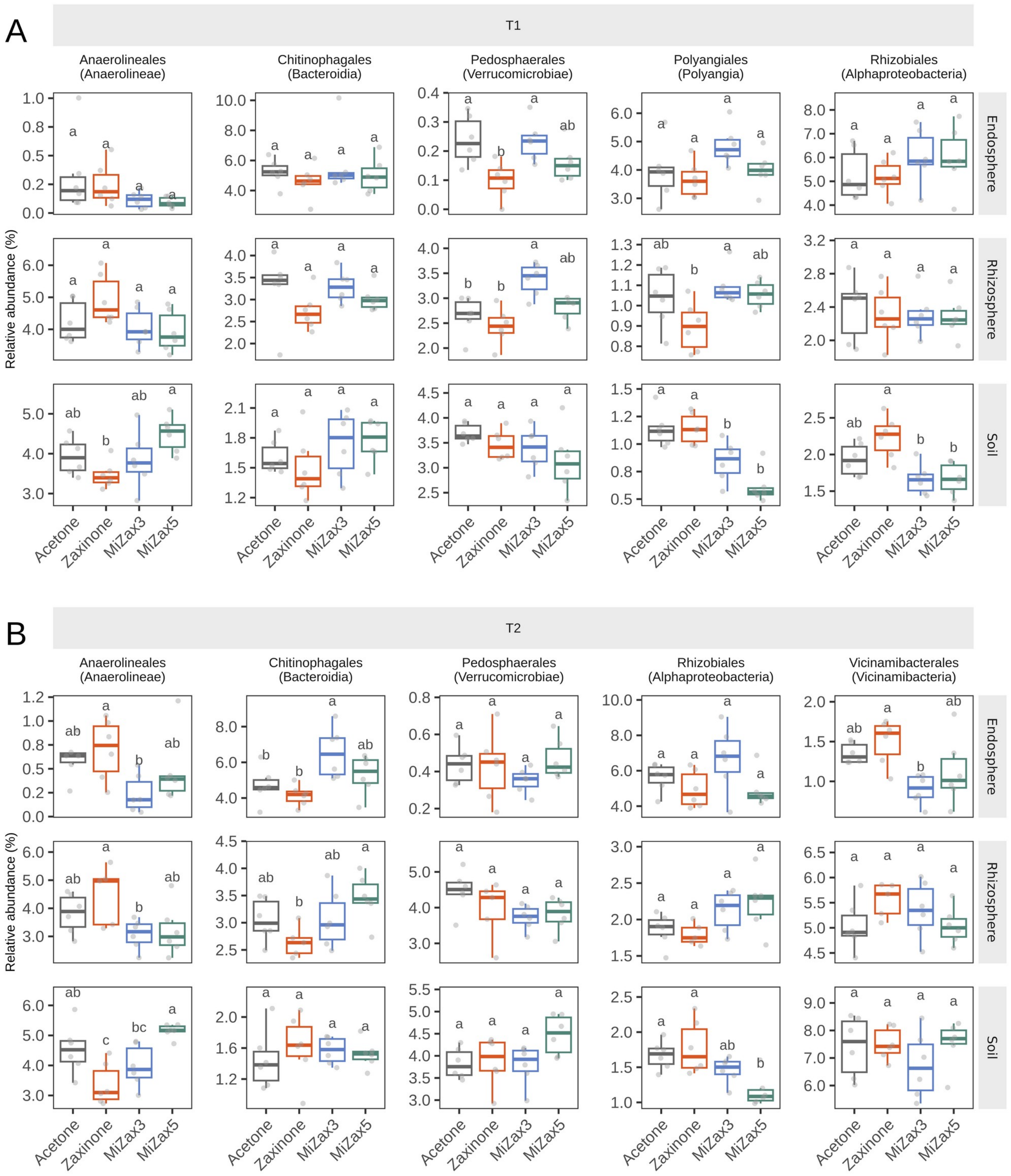

Figure S4. Relative abundance of selected bacterial orders in each treatment across timepoints and compartments. Timepoints are indicated in different panels (T1 in **A**, T2 in **B**) while different compartments are shown as rows. Below each order the corresponding class (upper taxonomic rank) is indicated. Boxplots display the median (horizontal line), the quartiles (boxes) and 1.5 interquartile range (whiskers), n = 6. Letters indicate statistically-supported differences between treatments (Tukey's post hoc test after ANOVA, $P<0.05$).

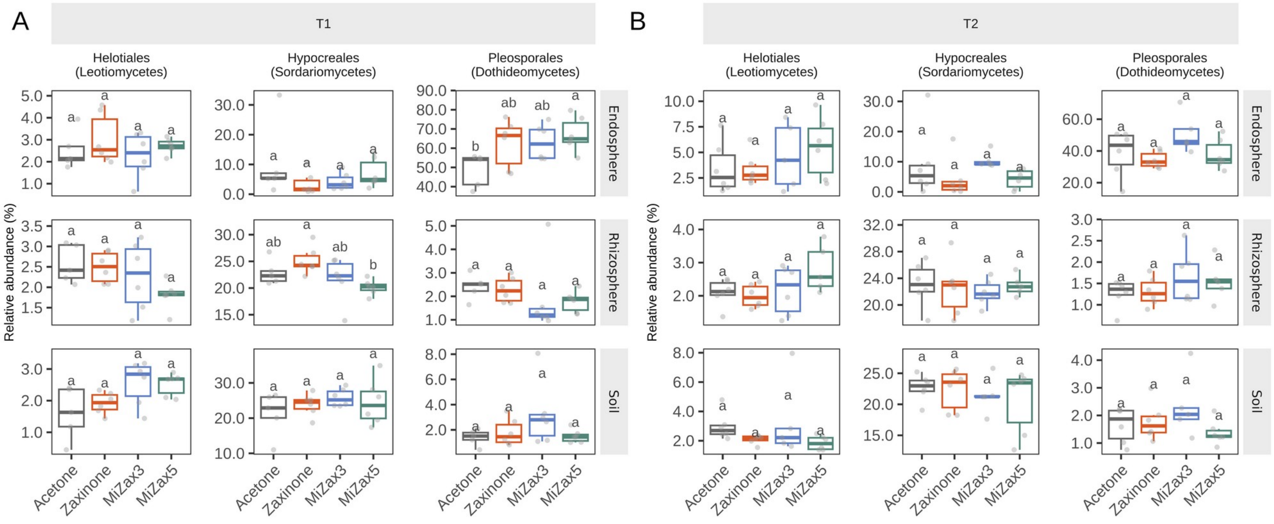

Figure S5. **Relative abundance of selected fungal orders in each treatment across timepoints and root compartment.** Timepoints are indicated in different panels (T1 in **A**, T2 in **B**) while different compartments are shown as rows. Below each order the corresponding class (upper taxonomic rank) is indicated. Boxplots display the median (horizontal line), the quartiles (boxes) and 1.5 interquartile range (whiskers), n = 6. Letters indicate statistically-supported differences between treatments (Tukey's posthoc test after ANOVA, P<0.05).

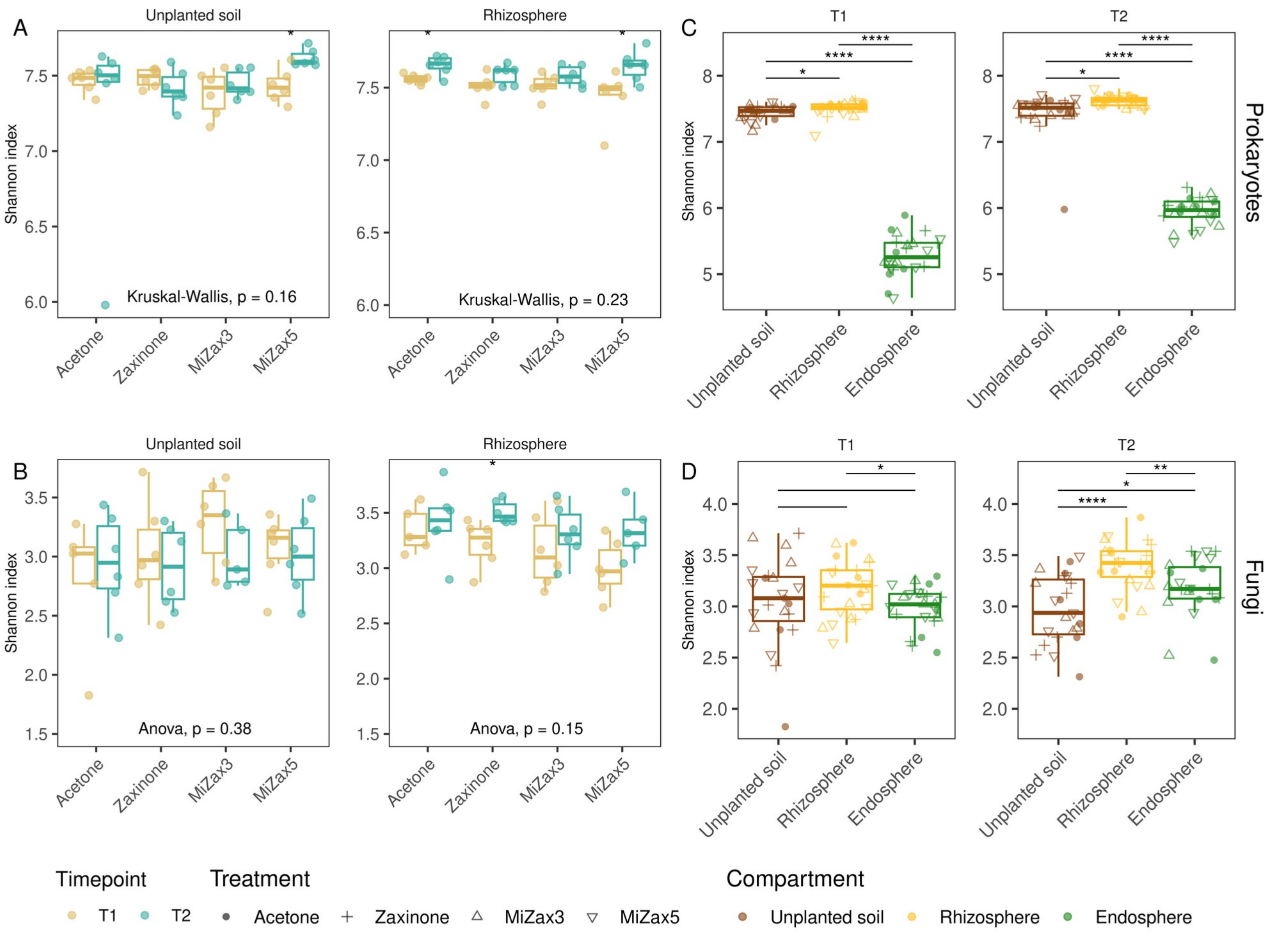

Figure S6. Shannon index values (α-diversity) grouped by compartment, treatment and timepoint in 16S and ITS2 amplicon libraries. (**A**, **B**) Shannon diversity index in prokaryotic (A) and fungal (B) libraries by treatments and timepoint in Unplanted soil and Rhizosphere compartments. (**C**, **D**) Comparison of the Shannon diversity index of the different compartments at both T1 and T2 in prokaryotic (C) and fungal (D) libraries. In each plot points represent single libraries, treatments are indicated with different shapes while root compartments and timepoints with different colours. T1, vegetative stage (60 days after transplanting); T2, milky stage maturation stage (120 days after transplanting). Boxplots display the median (horizontal line), the quartiles (boxes) and 1.5 interquartile range (whiskers). Asterisks indicate significant differences between means of the different timepoints (A, B) and compartments (C, D) according to the Student's t-test (*, $P < 0.05$; **, $P < 0.01$; ****, $P<0.0001$). No significant effect of the treatment was detected for both groups (Fungi and Prokaryotes) and compartments (unplanted soil and rhizosphere); the treatment p-value from Kruskal-Wallis/ANOVA analysis is indicated in the figure.

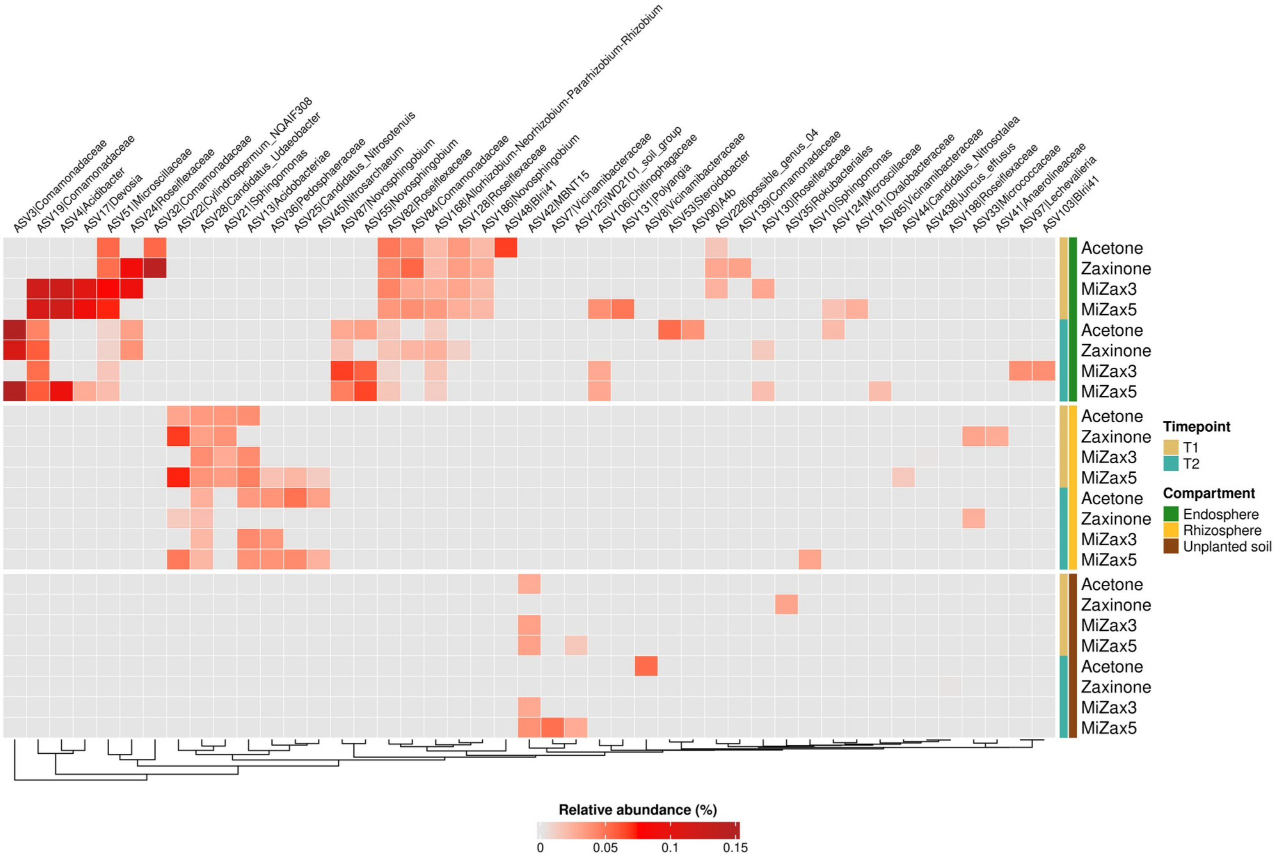

Figure S7. **Heatmap showing the relative abundances of compartment-enriched bacterial ASVs and their overlap across treatments and timepoints.** Only significantly enriched ASVs with a relative abundance higher than 2.5% in at least one condition were plotted. ASV number and the first meaningful taxonomic annotation available is indicated in columns.

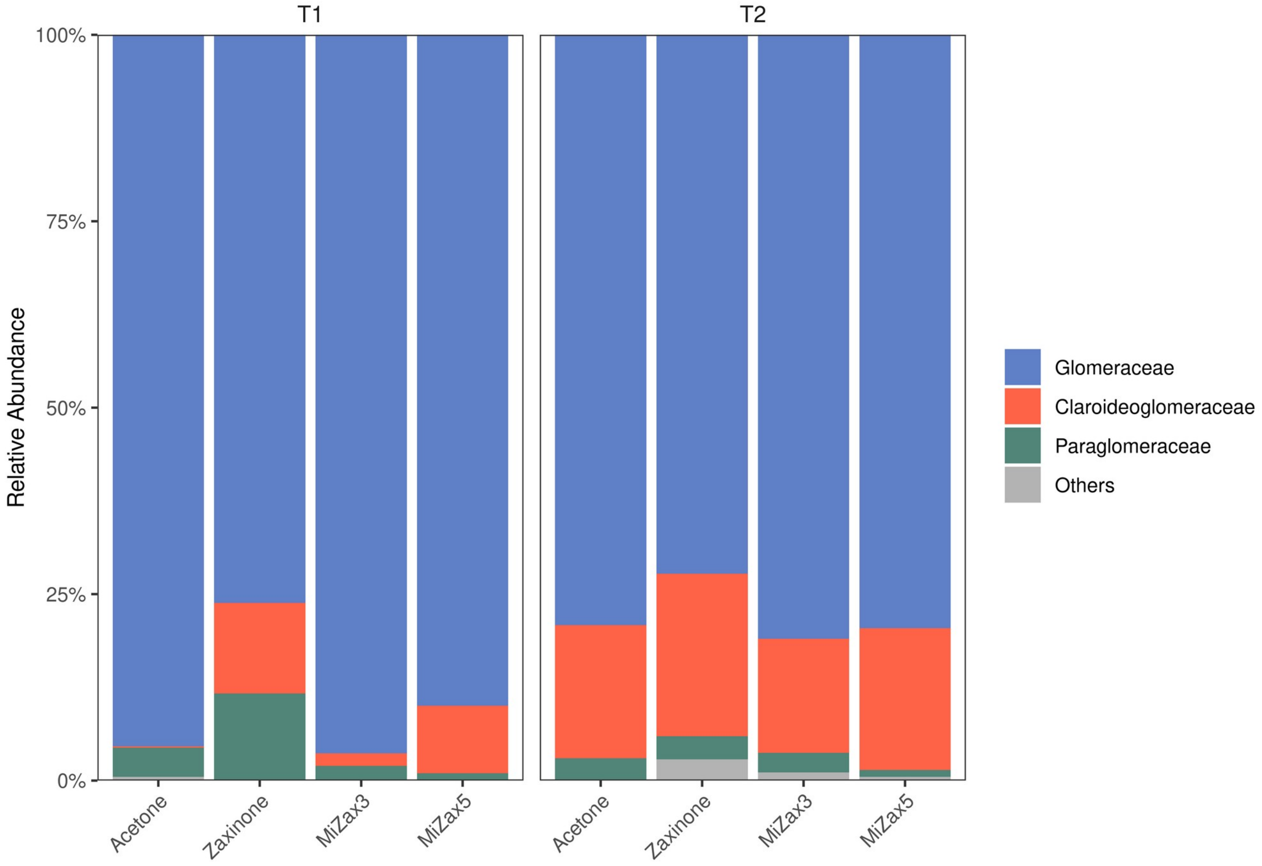

Figure S8. Assembly and diversity of root-associated microbiota under zaxinone and MiZax treatments at tillering (30 days after transplanting, T1) and milky-stage maturation (90 days after transplanting, T2). Average relative abundances of AMF community at the Class level.

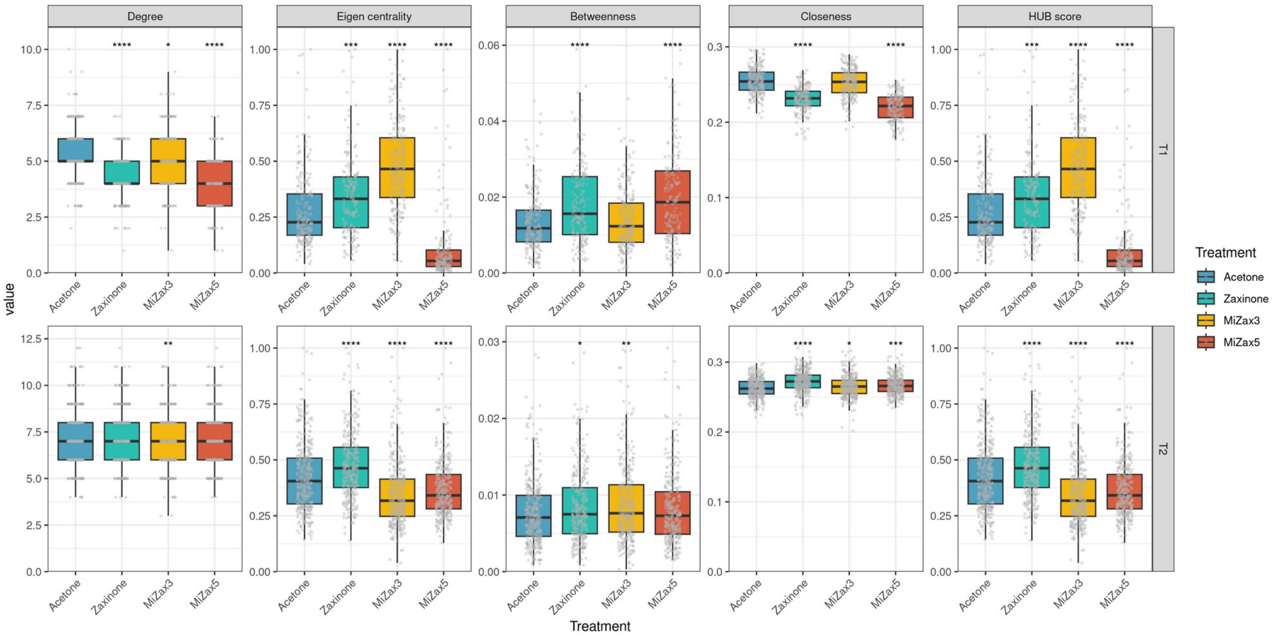

Figure S9. **Node centrality metrics of the root endosphere bacterial co-occurrence networks for each treatment and timepoint (rows).** Asterisks indicates statistically supported differences between each treatments and the acetone control treatment according to the Student's t-test (*P<0.05, **P<0.01, *** P<0.001, ****P<0.0001). Boxplots display the median (horizontal line), the quartiles (boxes) and 1.5 interquartile range (whiskers); points represent single nodes within each network.

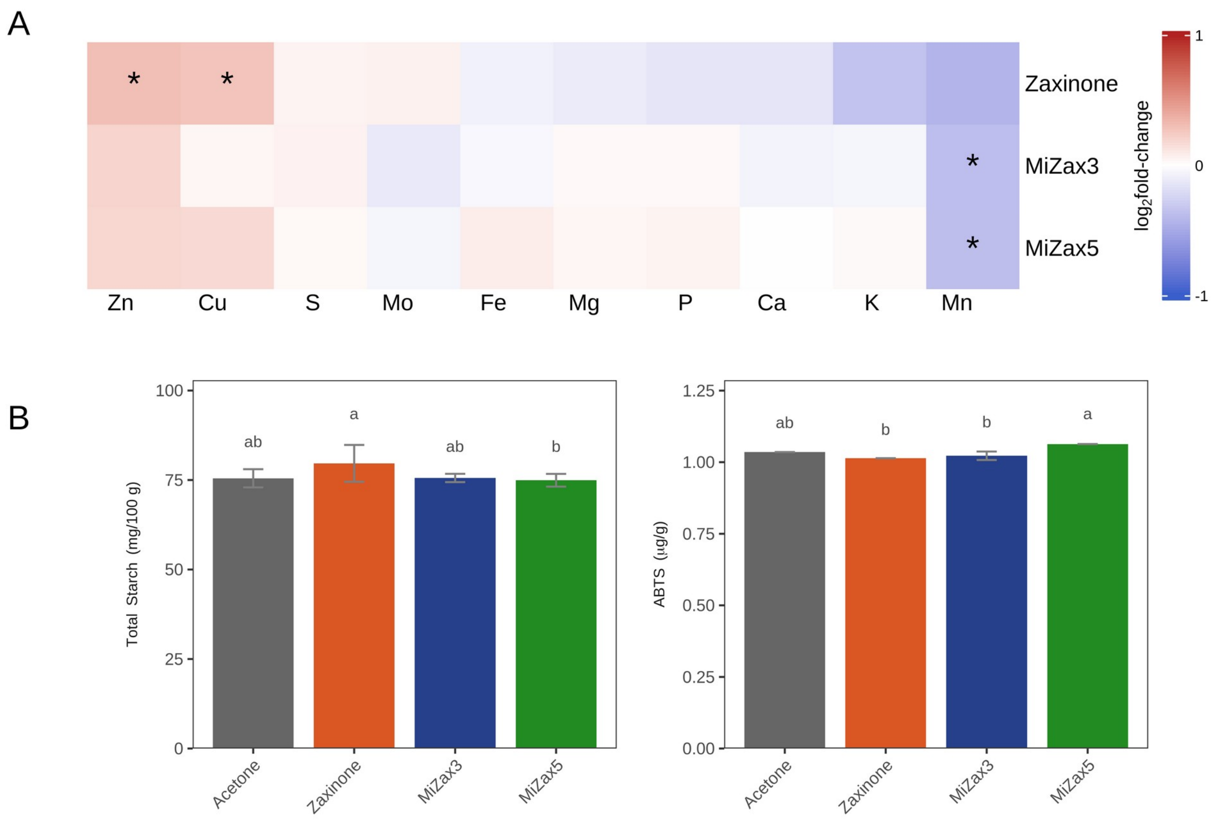

Figure S10. Analysis of biochemical parameters in rice grains from zaxinone and MiZax-treated plants. (A) Heatmap plot showing the log_2fold-change values of measured elements in each treatment compared to the acetone control NM. Blue and red colours depict a decrease and increase in metabolite levels respectively. Asterisks indicate statistically supported differences between each treatment and the reference acetone condition according to Dunn's post-hoc test after Kruskall-Wallis (* $P<0.05$, ** $P<0.01$, *** $P<0.001$). (B) Amount of total starch and antioxidant capacity (ABTS) measured on rice flour from the different treatments. ABTS values were expressed as the Trolox-equivalents per 100 g rice (μmol TE/100 g). Letters above each bar indicate statistically-supported differences across conditions according to Dunn's post-hoc test after Kruskall-Wallis ($P<0.05$).

Supplementary Tables

Table S1. Soil physico-chemical characteristics of the native paddy soil used in the mesocosm experiment. Values were measured on 3 soil subsamples, mean ± standard deviation for each parameter is indicated.

	measure unit	value
Sand	$g \cdot kg^{-1}$	473.22 ± 20.98
Silt	$g \cdot kg^{-1}$	426.50 ± 10.33
Clay	$g \cdot kg^{-1}$	100.28 ± 12.33
Texture	USDA classification	Loam
pH (H_2O)		7.77 ± 0.06
Total C (TC)	$g \cdot kg^{-1}$	14.15 ± 0.90
Total N (TN)	$g \cdot kg^{-1}$	1.73 ± 0.08
C/N ratio	ratio	8.59 ± 0.23
$CaCO_3$	$g \cdot kg^{-1}$	15.97 ± 0.82
P Olsen	$mg \cdot kg^{-1}$	48.75 ± 1.11
CEC*	$c\ mol^{+} \cdot kg^{-1}$	10.54 ± 0.77
**Exchangeable cations**		
Mg	$mg \cdot kg^{-1}$	153.33 ± 15.86
K	$mg \cdot kg^{-1}$	95.34 ± 2.26
Ca	$mg \cdot kg^{-1}$	1200.72 ± 68.09

Notes: *Cation Exchange Capability.

Table S2. PERMANOVA table (*adonis*, 9999 permutations) of the Bray-Curtis dissimilarities for bacterial and fungal microbial communities. Significant results ($p<0.05$) are highlighted in bold type.

**16S rDNA libraries (Prokaryotes)**						
**Source of variation**	**Df**	**Sums of Sqs.**	**R2**	**F**	**p-value**	**Explained variance (%)**
compartment	2	19.724	0.58866	144.4632	**0.0001**	58.866
treatment	3	0.466	0.0139	2.2743	**0.0188**	1.390
timepoint	1	1.446	0.04314	21.1757	**0.0001**	4.314
compartment × treatment	6	1.169	0.03489	2.8544	**0.0003**	3.489
compartment × timepoint	2	1.454	0.04341	10.6524	**0.0001**	4.341
treatment × timepoint	3	0.408	0.01216	1.9899	**0.0397**	1.216
compartment × treatment × timepoint	6	0.648	0.01934	1.5819	0.0552	1.934
Residual	120	8.192	0.24449			24.449
Total	143	33.507	1			100

**ITS2 rDNA libraries (Fungi)**						
**Source of variation**	**Df**	**Sums of Sqs.**	**R2**	**F**	**p-value**	**Explained variance (%)**
compartment	2	19.4752	0.6226	124.1137	**0.0001**	62.26
treatment	3	0.2457	0.00785	1.0438	0.3623	0.785
timepoint	1	0.5676	0.01814	7.234	**0.0006**	1.814
compartment × treatment	6	0.5179	0.01656	1.1002	0.3286	1.656
compartment × timepoint	2	0.8534	0.02728	5.4386	**0.0002**	2.728
treatment × timepoint	3	0.2378	0.0076	1.0105	0.3937	0.76
compartment × treatment × timepoint	6	0.4385	0.01402	0.9316	0.5114	1.402
Residual	114	8.9441	0.28593			28.593
Total	137	31.2803	1			100

Table S3. PERMANOVA table (*adonis*, 9999 permutations) of the Bray-Curtis dissimilarities for bacterial and fungal microbial communities grouped by compartment and timepoint. Significant results (p<0.05) are highlighted in bold type.

16S rDNA libraries (Prokaryotes)						
**Endosphere T1**	**Df.**	**Sums of Sqs.**	**R2**	**F**	***p-value***	**Explained variance (%)**
treatment	3	0.56	0.23	2.04	**0.001**	23.44
Residual	20	1.84	0.77			76.56
Total	23	2.4	1			100
**Endosphere T2**						
treatment	3	0.74	0.29	2.77	**0.0001**	29.36
Residual	20	1.77	0.71			70.64
Total	23	2.51	1			100
**Rhizosphere T1**						
treatment	3	0.24	0.18	1.42	0.07	17.52
Residual	20	1.11	0.82			82.48
Total	23	1.35	1			100
**Rhizosphere T2**						
treatment	3	0.35	0.25	2.2	**0.02**	24.84
Residual	20	1.05	0.75			75.16
Total	23	1.39	1			100
**Unplanted soil T1**						
treatment	3	0.37	0.25	2.17	**0.01**	24.6
Residual	20	1.13	0.75			75.4
Total	23	1.49	1			100
**Unplanted soil T2**						
treatment	3	0.44	0.25	2.27	**0.0003**	25.42
Residual	20	1.29	0.75			74.58
Total	23	1.73	1			100

ITS2 rDNA libraries (Fungi)						
**Endosphere T1**	**Df**	**Sums of Sqs.**	**R2**	**F**	**p-value**	**Explained variance (%)**
treatment	3	0.26	0.13	0.92	0.57	12.74
Residual	19	1.8	0.87			87.26
Total	22	2.07	1			100
**Endosphere T2**						
treatment	3	0.58	0.13	0.97	0.51	13.31
Residual	19	3.77	0.87			86.69
Total	22	4.35	1			100
**Rhizosphere T1**						
treatment	3	0.14	0.18	1.37	0.1	17.75
Residual	19	0.63	0.82			82.25
Total	22	0.77	1			100
**Rhizosphere T2**						
treatment	3	0.16	0.18	1.37	0.11	17.82
Residual	19	0.72	0.82			82.18
Total	22	0.88	1			100
**Unplanted soil T1**						
treatment	3	0.18	0.13	0.92	0.54	12.7
Residual	19	1.21	0.87			87.3
Total	22	1.39	1			100
**Unplanted soil T2**						
treatment	3	0.12	0.13	0.91	0.53	12.61
Residual	19	0.8	0.87			87.39
Total	22	0.92	1			100

Table S4. Pairwise PERMANOVA table (*adonis*. 9999 permutations) of the Bray-Curtis dissimilarities for the different prokaryotes communities. Significant results ($p<0.05$) are highlighted in bold type.

T1 - Unplanted soil						
**Pairs**	**Df**	**Sums of Sqs.**	**F. Model**	**R2**	**p-value**	**adjusted p-value**
Acetone vs MiZax3	1	0.0623337	1.084679	0.0978539	0.295	0.295
Acetone vs MiZax5	1	0.1304107	2.511379	0.2007276	0.003	**0.018**
Acetone vs Zaxinone	1	0.0835784	1.636398	0.1406275	0.102	0.153
MiZax3 vs MiZax5	1	0.0742791	1.206230	0.1076393	0.278	0.295
MiZax3 vs Zaxinone	1	0.1412228	2.325564	0.1886781	0.034	0.068
MiZax5 vs Zaxinone	1	0.2430916	4.404895	0.3057916	0.011	**0.033**

T2 - Unplanted soil						
**Pairs**	**Df**	**Sums of Sqs.**	**F. Model**	**R2**	**p-value**	**adjusted p-value**
Acetone vs MiZax3	1	0.0684950	0.8625125	0.0794027	0.585	0.585
Acetone vs MiZax5	1	0.1588905	2.5653276	0.2041592	0.013	**0.026**
Acetone vs Zaxinone	1	0.1663870	2.0603317	0.1708354	0.030	**0.045**
MiZax3 vs MiZax5	1	0.1393194	2.8803488	0.2236235	0.012	**0.026**
MiZax3 vs Zaxinone	1	0.0963320	1.4337556	0.1253967	0.145	0.174
MiZax5 vs Zaxinone	1	0.2506567	5.0420748	0.3351981	0.001	**0.006**

T1 - Rhizosphere						
**Pairs**	**Df**	**Sums of Sqs.**	**F. Model**	**R2**	**p-value**	**adjusted p-value**
Acetone vs MiZax3	1	0.0911381	1.356700	0.1194625	0.152	0.228
Acetone vs MiZax5	1	0.0755591	1.182208	0.1057222	0.205	0.246
Acetone vs Zaxinone	1	0.0862085	1.399001	0.1227302	0.050	0.228
MiZax3 vs MiZax5	1	0.0564236	1.133172	0.1017834	0.261	0.261
MiZax3 vs Zaxinone	1	0.0817088	1.720164	0.1467696	0.152	0.228
MiZax5 vs Zaxinone	1	0.0822477	1.859218	0.1567741	0.079	0.228

T2 - Rhizosphere						
**Pairs**	**Df**	**Sums of Sqs.**	**F. Model**	**R2**	**p-value**	**adjusted p-value**
Acetone vs MiZax3	1	0.0682804	1.2538995	0.1114191	0.190	0.228
Acetone vs MiZax5	1	0.0942010	1.8249212	0.1543284	0.092	0.138
Acetone vs Zaxinone	1	0.0986186	2.1024349	0.1737200	0.042	0.084
MiZax3 vs MiZax5	1	0.0436855	0.7553189	0.0702275	0.610	0.610
MiZax3 vs Zaxinone	1	0.1690871	3.1828297	0.2414375	0.016	**0.048**
MiZax5 vs Zaxinone	1	0.2186206	4.3472374	0.3030017	0.013	**0.048**

T1 - Endosphere						
**Pairs**	**Df**	**Sums of Sqs.**	**F. Model**	**R2**	**p-value**	**adjusted p-value**
Acetone vs MiZax3	1	0.1062069	1.154188	0.1034758	0.303	0.3030
Acetone vs MiZax5	1	0.1840651	1.947217	0.1629850	0.024	**0.0480**
Acetone vs Zaxinone	1	0.2358737	2.293669	0.1865732	0.025	**0.0480**
MiZax3 vs MiZax5	1	0.1378798	1.697408	0.1451097	0.058	0.0696
MiZax3 vs Zaxinone	1	0.2662671	2.973749	0.2292128	0.005	**0.0300**
MiZax5 vs Zaxinone	1	0.1970708	2.140963	0.1763421	0.032	**0.0480**

T2 - Endosphere						
**Pairs**	**Df**	**Sums of Sqs.**	**F. Model**	**R2**	**p-value**	**adjusted p-value**
Acetone vs MiZax3	1	0.2992602	3.373539	0.2522548	0.003	**0.006**
Acetone vs MiZax5	1	0.1491118	1.671478	0.1432105	0.021	**0.021**
Acetone vs Zaxinone	1	0.1612909	2.223456	0.1819008	0.002	**0.006**
MiZax3 vs MiZax5	1	0.2993760	2.860485	0.2224244	0.005	**0.006**
MiZax3 vs Zaxinone	1	0.3181702	3.615973	0.2655685	0.002	**0.006**
MiZax5 vs Zaxinone	1	0.2455352	2.774669	0.2172008	0.004	**0.006**

Table S5. Pairwise PERMANOVA table (*adonis*. 9999 permutations) of the Bray-Curtis dissimilarities for the different fungal communities. Significant results (p<0.05) are highlighted in bold type.

T1 - Unplanted soil						
**Pairs**	**Df**	**Sums of Sqs.**	**F. Model**	**R2**	**p-value**	**adjusted p-value**
Acetone vs MiZax3	1	0.0906293	1.1429582	0.11268489	0.256	0.732
Acetone vs MiZax5	1	0.053969	0.8198247	0.0834867	0.732	0.732
Acetone vs Zaxinone	1	0.05737572	0.7703198	0.07884285	0.725	0.732
MiZax3 vs MiZax5	1	0.03868185	0.7129326	0.06654878	0.706	0.732
MiZax3 vs Zaxinone	1	0.07454264	1.2014261	0.10725653	0.228	0.732
MiZax5 vs Zaxinone	1	0.03981954	0.7975424	0.07386333	0.58	0.732

T2 - Unplanted soil						
**Pairs**	**Df**	**Sums of Sqs.**	**F. Model**	**R2**	**p-value**	**adjusted p-value**
Acetone vs MiZax3	1	0.01686328	0.4497545	0.0475943	0.967	0.967
Acetone vs MiZax5	1	0.05693306	1.3093455	0.11577553	0.213	0.6975
Acetone vs Zaxinone	1	0.03812559	0.837483	0.07727652	0.465	0.6975
MiZax3 vs MiZax5	1	0.03739026	0.9696255	0.09725797	0.388	0.6975
MiZax3 vs Zaxinone	1	0.02686668	0.6580071	0.06813074	0.681	0.8172
MiZax5 vs Zaxinone	1	0.05247284	1.1288256	0.10143259	0.294	0.6975

T1 - Rhizosphere						
**Pairs**	**Df**	**Sums of Sqs.**	**F. Model**	**R2**	**p-value**	**adjusted p-value**
Acetone vs MiZax3	1	0.04229463	1.145.755	0.11292952	0.284	0.3408
Acetone vs MiZax5	1	0.05494233	2.225.339	0.19824248	0.05	0.195
Acetone vs Zaxinone	1	0.04849414	172.137	0.160555	0.065	0.195
MiZax3 vs MiZax5	1	0.04940456	1.311.273	0.11592623	0.224	0.336
MiZax3 vs Zaxinone	1	0.03911445	0.95843	0.08746052	0.401	0.401
MiZax5 vs Zaxinone	1	0.03860308	1.295.029	0.11465475	0.207	0.336

T2 - Rhizosphere						
**Pairs**	**Df**	**Sums of Sqs.**	**F. Model**	**R2**	**p-value**	**adjusted p-value**
Acetone vs MiZax3	1	0.0385935	0.8635826	0.07949335	0.488	0.5856
Acetone vs MiZax5	1	0.04202185	11.772.037	0.11567065	0.267	0.4005
Acetone vs Zaxinone	1	0.05821635	14.237.313	0.12462927	0.105	0.244
MiZax3 vs MiZax5	1	0.02925792	0.8371146	0.08509757	0.62	0.62
MiZax3 vs Zaxinone	1	0.06549974	16.285.725	0.14004922	0.122	0.244
MiZax5 vs Zaxinone	1	0.07972768	25.945.684	0.22377447	0.014	0.084

T1 - Endosphere						
**Pairs**	**Df**	**Sums of Sqs.**	**F. Model**	**R2**	**p-value**	**adjusted p-value**
Acetone vs MiZax3	1	0.09168269	0.8074981	0.08233477	0.643	0.7716
Acetone vs MiZax5	1	0.13038263	12.819.713	0.12468147	0.215	0.735
Acetone vs Zaxinone	1	0.11048722	0.9837321	0.0985335	0.445	0.735
MiZax3 vs MiZax5	1	0.07543864	0.9513652	0.08687184	0.451	0.735
MiZax3 vs Zaxinone	1	0.04887284	0.550099	0.05214159	0.847	0.847
MiZax5 vs Zaxinone	1	0.07549176	0.9654572	0.08804532	0.49	0.735

T2 - Endosphere						
**Pairs**	**Df**	**Sums of Sqs.**	**F. Model**	**R2**	**p-value**	**adjusted p-value**
Acetone vs MiZax3	1	0.1499497	0.7060818	0.07274633	0.853	0.869
Acetone vs MiZax5	1	0.1302824	0.6997238	0.06539643	0.869	0.869
Acetone vs Zaxinone	1	0.1666122	0.7696294	0.07146294	0.78	0.869
MiZax3 vs MiZax5	1	0.2101296	11.771.836	0.11566889	0.262	0.786
MiZax3 vs Zaxinone	1	0.3253509	15.335.151	0.14558437	0.111	0.666
MiZax5 vs Zaxinone	1	0.1841853	0.9902245	0.09010048	0.441	0.869

Table S6. Network-level metrics for each of the bacterial community co-occurrences networks constructed. Asterisks indicate differences between real co-occurrence networks and their associated random networks (999 permutations, Z-test, P<0.05).

Networks	Nodes	Edges	Positive edges (%)	Transitivity	degree	path length	Betweenness centrality (normalized)	Closeness centrality (normalized)	Eigen-centrality (normalized)	connectivity	Modularity	HUBs No.	Modules No.
ACE_T1_endo	222	596	67.45	0.13*	5.37	3.94*	0.013*	0.255*	0.28*	2	0.51*	6	8*
ZAX_T1_endo	188	401	60.10	0.13*	4.27	4.36*	0.018*	0.231*	0.35*	1	0.58*	1	9*
MX3_T1_endo	215	550	56.55	0.12*	5.12	3.99*	0.014*	0.252*	0.48*	1	0.53*	2	8*
MX5_T1_endo	186	364	60.44	0.10*	3.91	4.59*	0.020*	0.219*	0.11*	1	0.61*	1	10*
ACE_T2_endo	363	1305	62.53	0.17*	7.19	3.81*	0.008*	0.263*	0.42*	4	0.51*	10	6*
ZAX_T2_endo	317	1147	56.58	0.17*	7.24	3.69*	0.009*	0.272*	0.48*	4	0.48*	7	7*
MX3_T2_endo	316	1084	64.85	0.18*	6.86	3.78*	0.009*	0.265*	0.35*	3	0.51*	8	6*
MX5_T2_endo	338	1190	60.67	0.16*	7.04	3.76*	0.008*	0.267*	0.37	4	0.51*	10	7*

Supplementary Datasets (Provided as separate Excel or tab-delimited files)

Dataset S1. Raw reads count and ASVs calling statistics for 16S and ITS2 rDNA amplicon libraries.
Dataset S2. Differentially abundant prokaryotic ASVs across treatments, timepoints and compartments.
Dataset S3. Differentially abundant fungal ASVs across treatments, timepoints and compartments.
